# Chronic stress‐induced brain‐derived small extracellular vesicles promote colorectal cancer through Treg differentiation

**DOI:** 10.1002/ctm2.70756

**Published:** 2026-08-21

**Authors:** Huanhe Ni, Yunyan Ling, Ting Tang, Yuyang Liu, Zhen Chai, Shunjie Qing, Xujie Xiao, Qiqiong Ding, Dandan Zhao, Hui Pan, Yan Lin, Dehua Wu

**Affiliations:** ^1^ Department of Radiation Oncology, Nanfang Hospital Southern Medical University Guangzhou China; ^2^ Department of Laboratory Ganzhou People's Hospital Ganzhou China; ^3^ Department of Gynaecology Ganzhou People's Hospital Ganzhou China; ^4^ Department of General Surgery Nanfang Hospital Southern Medical University Guangzhou China; ^5^ Clinical Pharmacology Research Center, Peking Union Medical College Hospital, State Key Laboratory of Complex Severe and Rare Diseases, NMPA Key Laboratory for Clinical Research and Evaluation of Drug, Beijing Key Laboratory of Clinical PK & PD Investigation for Innovative Drugs Chinese Academy of Medical Sciences & Peking Union Medical College Beijing China; ^6^ Guangdong Provincial Key Laboratory for Prevention and Control of Major Liver Diseases Guangzhou China

**Keywords:** brain‐derived sEVs (BD‐sEVs), chronic stress, colorectal cancer (CRC), NOXA, regulatory T cells (Treg)

## Abstract

**Background:**

Chronic stress is increasingly recognised as a risk factor for poor prognosis in colorectal cancer (CRC) through sustained activation of the hypothalamic–pituitary–adrenal (HPA) axis and sympathetic nervous system, yet the mechanisms by which psychological stress signals are transmitted from the central nervous system to the peripheral tumour immune microenvironment remain poorly understood. Brain‐derived small extracellular vesicles (BD‐sEVs), which can cross the blood–brain barrier, represent potential mediators of this neuroimmune crosstalk.

**Methods:**

A chronic restraint stress (CRS) mouse model was established in both subcutaneous (MC38) and orthotopic (CT26) CRC models. BD‐sEVs were enriched from plasma using anti‐L1 cell adhesion molecule (L1CAM) antibodies and characterised by transmission electron microscopy and nanoparticle tracking analysis. Multichannel flow cytometry was used to analyse immune cell populations in the tumour microenvironment. Mechanistic studies included microRNA (miRNA) sequencing, single‐cell RNA sequencing, co‐immunoprecipitation, mass spectrometry, mitochondrial function assessment and mitochondrial DNA (mtDNA) detection. Clinical validation was performed in a retrospective cohort (*n* = 67) and a prospective cohort (*n* = 37) of CRC patients, with anxiety levels assessed by the Hamilton Anxiety Rating Scale (HAMA).

**Results:**

We show that chronic stress‐activated brain regions remodel the miRNA cargo of circulating BD‐sEVs, which are taken up by CD4^+^ naïve T cells within the tumour microenvironment, promoting regulatory T‐cell (Treg) differentiation and immunosuppressive function. Mechanistically, stress‐responsive miRNAs (miR‐342‐3p, miR‐15a‐5p and miR‐381‐3p) upregulate phorbol‐12‐myristate‐13‐acetate‐induced protein 1 (NOXA), which directly binds the mitochondrial chaperone heat shock protein 60 (HSP60) and inhibits its chaperone activity, triggering selective mtDNA release into the cytoplasm. Cytosolic mtDNA activates the cyclic GMP‒AMP synthase (cGAS)‒stimulator of interferon genes (STING)‒type I interferon (IFN‐I) pathway, driving Treg differentiation independently of the canonical myeloid cell leukaemia 1 (MCL‐1)‐dependent apoptotic pathway. Clinically, intratumoural CD4^+^NOXA/Forkhead box P3 (FOXP3) expression correlates significantly with patient anxiety scores and predicts adverse survival outcomes.

**Conclusion:**

Our study reveals an immunosuppressive ‘chronic stress‒BD‐sEV‒NOXA‒HSP60‒Treg’ axis and provides crucial mechanistic insights into the psychoneuroimmunological contributions to cancer progression and novel targets for therapeutic intervention.

**Key points:**

- Chronic stress promotes the differentiation of regulatory T cells (Treg) in the tumour microenvironment through brain‐derived small extracellular vesicles (BD‐sEVs) to inhibit antitumour immunity.
- Mechanistically, BD‐sEV‐induced phorbol‐12‐myristate‐13‐acetate‐induced protein 1 (NOXA) binds heat shock protein 60 (HSP60) and inhibits its chaperone activity, triggering mitochondrial DNA (mtDNA) release and activation of the cyclic GMP‒AMP synthase (cGAS)‒stimulator of interferon genes (STING) pathway to drive Treg differentiation.
- The stress‒BD‐sEV‒NOXA‒HSP60‒Treg axis is a key pathway that connects psychological stress with peripheral tumour immunosuppression.
- The expression of NOXA and Forkhead box P3 (FOXP3) in clinical tumour samples correlates with the level of anxiety and may serve as potential prognostic biomarkers.

## Graphical abstract

Chronic stress‐induced BD‐sEVs act as central‐to‐peripheral mediators, conveying stress signals to colorectal tumours and driving Treg differentiation via NOXA/HSP60‐triggered mtDNA release and cGAS‒STING activation, which suppresses antitumour immunity and correlates with anxiety and prognosis.

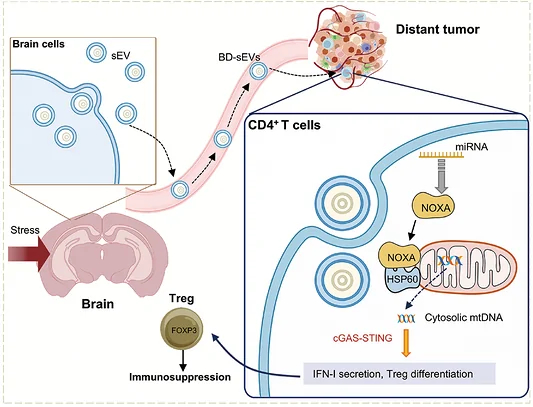

## 1 INTRODUCTION

Colorectal cancer (CRC) is a common malignancy of the digestive system whose prognosis is increasingly recognised to be influenced by chronic stress.^1^, ^2^ Prolonged activation of stress‐associated pathways—the hypothalamic–pituitary–adrenal (HPA) axis and the sympathetic nervous system (SNS)—impairs immune surveillance, metabolic regulation and cellular integrity, thereby precipitating a range of pathological conditions including tumourigenesis and immune dysregulation.^3^, ^4^, ^5^, ^6^ Accumulating epidemiological and experimental evidence confirms that chronic stress promotes the progression, metastasis and immune evasion of multiple malignancies,^6^, ^7^, ^8^, ^9^ with emerging data supporting extensive regulation of the mammalian immune system by the central nervous system (CNS).^1^ During CRC progression, stress‐induced immune dysregulation alters the function and differentiation of key immune cell types, including antigen‐presenting cells, natural killer cells and regulatory T cells (Treg), within the tumour microenvironment (TME).^4^, ^10^ However, the mechanisms by which peripheral immune cells perceive neural signals from the chronically stressed brain—across the blood–brain barrier—to mediate immunosuppression and tumour progression remain largely unexplored.

Brain‐derived small extracellular vesicles (BD‐sEVs) are L1 cell adhesion molecule (L1CAM)‐positive nanoscale extracellular vesicles (30–150 nm) secreted by neurons and glial cells that carry brain‐specific cargo, including proteins, nucleic acids and lipids.^11^, ^12^, ^13^ These specialised vesicles can cross the blood–brain barrier via transcytosis, establishing a direct channel of communication between the CNS and peripheral tissues.^11^, ^14^ Chronic stress activates specific brain regions associated with small extracellular vesicle (sEV) secretion, including the hippocampal CA1–CA3 areas and the caudate putamen (CPu), thereby altering the molecular composition and functional capacity of BD‐sEVs.^8^, ^15^ Additionally, stress‐induced changes in the content of BD‐sEVs have been observed in patients with neurodegenerative diseases such as Alzheimer's disease and Parkinson's disease.^16^ Notably, under certain disease conditions, BD‐sEVs can carry immunosuppressive molecules (such as programmed death‐ligand 1 [PD‐L1] or FasL) to prevent overactivation of the immune system by directly inhibiting the activity of peripheral immune cells such as effector T cells. While this immunosuppressive capacity serves a homeostatic role in preventing excessive inflammation, the same molecular machinery can be co‐opted in pathological contexts to promote immune evasion. In addition, this immunosuppressive mechanism has also been observed in the context of neurological diseases (such as multiple sclerosis) and TMEs.^7^, ^17^ However, the critical role of stress‐altered BD‐sEV molecular cargo in regulating immune cell differentiation and function requires further elucidation.

Phorbol‐12‐myristate‐13‐acetate‐induced protein 1 (NOXA), encoded by the PMAIP1 gene, is traditionally recognised as a pro‐apoptotic BH3‐only protein in the BCL‐2 family that functions in p53‐dependent apoptotic pathways.^18^ In addition to its role in apoptosis, NOXA is involved in DNA damage repair, cell cycle regulation, metabolic reprogramming and antiviral immune responses.^18^ NOXA protein stability is tightly regulated by the ubiquitin‒proteasome system through the WSB2‐containing CRL5 E3 ubiquitin ligase complex.^19^ NOXA can promote cell growth by supporting proliferative metabolism in activated T cells, and its expression is crucial for cell survival under glucose‐restricted conditions.^19^ However, the nonapoptotic functions of NOXA, particularly its role in immune cell differentiation within the TME and its potential posttranslational modifications involved in cancer progression, remain poorly understood.

Treg inhibit antitumour immunity through a variety of mechanisms, including the secretion of immunosuppressive (cytokines interleukin‐10 [IL‐10] and transforming growth factor‐β [TGF‐β]) and the direct inhibition of effector T cells by cytotoxic T‐lymphocyte‐associated protein 4 (CTLA‐4) and membrane‐bound TGF‐β.^20^ Our previous studies revealed that cytoplasmic DNA promoted Treg differentiation through the cyclic GMP‒AMP synthase (cGAS)‒stimulator of interferon genes (STING) signalling pathway.^21^ Recent studies have revealed that tumour‐derived sEVs (TDEs) can activate the cGAS‒STING pathway, promoting Treg differentiation.^22^, ^23^ Furthermore, STING trafficking from the endoplasmic reticulum to the Golgi activates mitogen‐activated protein kinase–cAMP response element‐binding protein (MAPK‐CREB) signalling to induce IL‐2 and TGF‐β2 expression, thereby facilitating Treg differentiation independently of type I interferon (IFN‐I).^24^ As critical components of the immunosuppressive TME, Treg exhibit increased activity and differentiation under chronic stress conditions in mammals.^10^, ^25^ Both HPA axis activation (via cortisol) and SNS signalling (via norepinephrine) directly promote Treg differentiation and suppressive function, demonstrating the profound neuroendocrine regulation of these cells.^26^ However, whether Treg are regulated by stress‐induced BD‐sEVs (Stress‐BD‐sEVs) to suppress antitumour immunity remains unexplored.

Here, we investigate whether Stress‐BD‐sEVs regulate CD4^+^ T‐cell differentiation to suppress antitumour immunity. We specifically test the hypothesis that BD‐sEV‐derived microRNAs (miRNAs) upregulate the BH3‐only protein NOXA, driving Treg differentiation through a mitochondrial‐dependent mechanism. Our findings reveal a brain‐to‐tumour immunosuppressive axis that provides a mechanistic framework connecting psychoneural factors with tumour immune escape.

## 2 MATERIALS AND METHODS

### 2.1 Human participants

CRC patients who underwent surgical resection at Nanfang Hospital, Southern Medical University were enrolled in this study. All procedures were approved by the Medical Ethics Committee of Nanfang Hospital, Southern Medical University (NFEC‐2023‐465). The retrospective cohort included 67 CRC patients whose formalin‐fixed paraffin‐embedded (FFPE) tumour specimens and clinical data were collected for immunohistochemistry (IHC) and prognostic analysis (Table S1). Tumour staging was performed according to the 8th edition of the AJCC staging system. Patients who received neoadjuvant chemotherapy or radiotherapy prior to surgery were excluded. For survival analysis, patients were stratified into ‘high’ and ‘low’ expression groups using the median value as the cutoff. A prospective cohort of 37 patients underwent anxiety assessment using the Hamilton Anxiety Rating Scale (HAMA), and tumour marker expression was evaluated by IHC (Table S2). The privacy rights of human subjects were observed, and informed consent was obtained for experimentation with human subjects.

### 2.2 Mice

C57BL/6 mice (8–12 weeks old, male) were purchased from Risemice Biotechnology Co., Ltd.  All animal experiments were approved by the Animal Care and Use Committee of Southern Medical University (SMUL2022189) and conducted in accordance with the Guide for the Care and Use of Laboratory Animals, with humane endpoints set at a maximum tumour volume of 2000 mm^3^ or a maximum tumour diameter of 20 mm.

### 2.3 Chronic restraint stress model

The mice were randomly divided into chronic restraint stress (CRS) and control groups. CRS mice were placed in 50 mL centrifuge tubes with ventilation holes, where they were restrained from 9:00 a.m. to 3:00 p.m. (6 h/day), after which they were released and given access to food and water. The control mice were food‐ and water‐matched (pair‐fed) for the same period but allowed to move freely. In the MC38 subcutaneous model, the CRS protocol lasted 21 consecutive days (day 1 to day 21). MC38 cells (1 × 10^6^) were inoculated subcutaneously on day 7, and CRS continued through day 21, with endpoint at day 24. Open field tests (OFT) were conducted at days 7 (pre‐inoculation), 14 and 21 to confirm sustained anxiety‐like behaviour throughout the experimental period.

### 2.4 Open field test

The mice were habituated to the testing room for 30 min before testing began. The open field arena (50 cm × 50 cm × 40 cm) was used to record the movement trajectories of each mouse for 5 min. After each test, faeces/urine were removed, and the arena was cleaned with 75% ethanol to eliminate residual odours. Reductions in the time spent or distance travelled in the central zone indicated increased anxiety.

### 2.5 Sucrose preference test

The mice were habituated to two bottles, one containing 1% sucrose solution and the other containing tap water, for at least 2 days, and their positions were alternated every 12 h to avoid side bias. Sucrose preference was calculated as follows: [sucrose intake/(sucrose + water intake)] × 100%, and the results were averaged over the testing period to evaluate anhedonia.

### 2.6 Isolation and identification of sEVs

In accordance with the MISEV2023 guidelines, these L1CAM‐enriched vesicles are hereafter referred to as BD‐sEVs, acknowledging that the isolated particles represent an enriched population of L1CAM‐positive sEVs without specific claims about their endosomal biogenesis pathway.^27^ Mouse blood was collected via retro‐orbital bleeding into EDTA‐coated tubes and centrifuged (1500 × *g*, 15 min, 4°C) to obtain plasma. sEVs were isolated from mouse brains/plasma using an ExoQuick precipitation kit (System Biosciences) per the manufacturer's instructions. We acknowledge that precipitation‐based methods can co‐isolate non‐EV components; however, the subsequent L1CAM immunoprecipitation step provides additional purification specificity for BD‐sEV enrichment. To enrich blood‐derived BD‐sEVs, plasma sEV resuspensions were incubated with 2 µg of biotinylated anti‐mouse L1CAM antibody (Origene, OTI3G1) in 3% bovine serum albumin (BSA)for 60 min at 20°C, followed by the addition of 10 µL of Streptavidin Plus UltraLink resin. After centrifugation (400 × *g*, 10 min, 4°C), the pellets were washed with 0.05 M glycine‒HCl (pH 3.0) supplemented with 3% BSA and then eluted with 1 M Tris‒HCl (pH 8.0) containing M‐PER supplemented with protease/phosphatase inhibitors.^28^, ^29^ sEV morphology was observed via transmission electron microscopy (TEM; JEM1230/Olympus EM208S), and their size distribution and concentration were analysed by nanoparticle tracking analysis (NTA; NanoSight NS300), with each sample tested in triplicate.

To assess the potential contribution of peripheral nerve‐derived sEVs (PNS‐sEVs), sEVs were enriched in parallel from the same plasma samples using anti‐p75‐NTR antibodies (a marker for peripheral nerve‐derived vesicles).^30^ Plasma sEV resuspensions were incubated with 2 µg of biotinylated anti‐mouse p75‐NTR antibody in 3% BSA for 60 min at 20°C, followed by Streptavidin Plus UltraLink resin capture, washing and elution under identical conditions to the L1CAM protocol. PNS‐sEV protein mass was quantified by BCA assay, and sEV marker expression (CD63, TSG101, L1CAM and p75‐NTR) was verified by Western blotting.

### 2.7 BD‐sEV‐treated animal model

Mice were subcutaneously inoculated with MC38 cells to establish CRC models, followed by tail vein injection of control BD‐sEVs (Ctrl‐BD‐sEVs) or Stress‐BD‐sEVs every 3 days for 2 weeks. Approximately 18 days after MC38 cell inoculation, the mice were euthanised, and tumour tissues were collected for flow cytometry or IHC.^31^, ^32^

### 2.8 T‐cell activation and differentiation in vitro

Human naïve T cells were isolated from peripheral blood mononuclear cells (PBMCs) obtained from healthy donors via negative selection (Miltenyi Biotech). Murine naïve T cells were isolated from mouse spleens/lymph nodes using the same method. Cells were seeded on αCD3/αCD28‐precoated plates (BioLegend) in medium supplemented with IL‐2 (100 IU/L; PeproTech) and cultured for 3 days. The antibodies and cytokines used are listed in Table S4. For BD‐sEV treatment, T cells were transfected with short hairpin RNA (shRNA) targeting NOXA (sh‐NOXA), heat shock protein 60 (HSP60; sh‐HSP60), or control viruses within the first 12 h and then treated with BD‐sEVs. The proportions of Treg were determined by flow cytometry or Western blotting.

### 2.9 Flow cytometry

Cells were stained with Zombie NIR viability dye (BioLegend) and surface antibodies and then fixed/permeabilised with a Foxp3 Fixation/Permeabilisation Kit (eBioscience) for intracellular staining. Flow cytometry was performed using a CytExpert flow cytometer (BD), and the antibodies used are listed in Table S4. The data were analysed with FlowJo v10.6.2.

### 2.10 T‐cell proliferation and Treg suppression assays

T cells were labelled with carboxyfluorescein succinimidyl ester (CFSE) (Invitrogen) per the manufacturer's instructions and cultured for 3 days, after which proliferation was analysed by flow cytometry. For the Treg suppression assays, CD4^+^CD25^+^ Treg were isolated from among treated T cells, and CFSE‐labelled naïve T cells (5 × 10^4^/well) were cocultured with Treg at a 1:10 ratio in αCD3/αCD28‐precoated 96‐well plates. After 5 days, CFSE^+^ T‐cell proliferation was detected by flow cytometry.

### 2.11 Plasmid construction, lentivirus production and transduction

sh‐NOXA/sh‐HSP60 plasmids were constructed by cloning the shRNA sequences and inserting them into the pLKO.1 vector (AgeI/EcoRI sites). Full‐length NOXA/HSP60 genes with C‐terminal His/Flag tags were subcloned and inserted into the pSin vector (BamHI/EcoRI sites). HEK293T cells (70% confluence) were transfected with the target plasmids psPAX2 and pMD2.G (4:3:1 ratio) and PEI (64 µg/mL in Opti‐MEM) for 6 h and then cultured for 48 h. Lentiviral supernatants were filtered (0.45 µm) and centrifuged (100 000 × *g*, 3 h, 4°C), and the pellets were resuspended in RPMI 1640. T cells were infected with lentiviruses in the presence of 8 µg/mL polybrene (Sigma–Aldrich). The reagents used are listed in Table S5.

### 2.12 Mass spectrometry analysis

Cells were lysed on ice for 1 h with IP lysis buffer (Beyotime) and incubated overnight at 4°C with primary antibodies, after which the target proteins were pulled down with protein A/G beads. The immunoprecipitated samples were sent to Jingjie PTM BioLab (Hangzhou) for mass spectrometry (MS) analysis.

### 2.13 Measurement of mitochondrial function

Reactive oxygen species (ROS) levels were detected with 10 µm2′,7′‐dichlorodihydrofluorescein diacetate (DCFH‐DA) (Beyotime) via flow cytometry. Adenosine triphosphate (ATP) levels were measured using the Cell Titer‐Glo Luminescent assay (Promega) and normalised to protein mass. The mitochondrial membrane potential was assessed with JC‐1 dye (Beyotime). Briefly, the cells were stained with 5 mg/L JC‐1 for 20 min and then analysed by flow cytometry.

### 2.14 Subcellular fractionation and cytosolic mitochondrial DNA detection

CD4^+^ naïve T cells (3 × 10^6^) were resuspended in digitonin buffer (150 mM NaCl, 50 mM HEPES [pH 7.4], 25 µg/mL digitonin; EMD Millipore), incubated for 10 min at room temperature, and centrifuged (16 000 × *g*, 25 min, 4°C) to separate cytosolic and mitochondrial fractions.^23^, ^33^ The cytosolic fraction was analysed by Western blotting (cytochrome C, COX IV, β‐actin) and quantitative PCR (qPCR) for mitochondrial DNA (mtDNA) (ND1 and ND5) using the Human Mitochondrial DNA Monitoring Primer Set (Takara). The mitochondrial fraction was verified by COX IV positivity.

### 2.15 Multicolour immunofluorescence staining

FFPE tumour sections from CRC patients were subjected to four‐colour immunofluorescence staining: DAPI (nuclear), CD4 (Alexa Fluor 647), FOXP3 (Alexa Fluor 488) and NOXA (Alexa Fluor 594). Full‐slide fluorescence scans were acquired. Single‐stain controls for each fluorophore were acquired to verify channel specificity and the absence of spectral cross‐talk. Quantitative analysis of NOXA^+^ cells within gated CD4^+^FOXP3^+^ Treg cells was performed using ImageJ. For each patient, the following parameters were quantified: (i) total CD4^+^FOXP3^+^ Treg cell count per mm^2^ tumour tissue; (ii) CD4^+^FOXP3^+^NOXA^+^ triple‐positive cell count per mm^2^; and (iii) mean fluorescence intensity (MFI) of NOXA within the gated CD4^+^FOXP3^+^ Treg population. Pearson correlation analysis was performed using patient‐level summary statistics.

### 2.16 Single‐cell RNA sequencing data acquisition and analysis

To identify conserved Treg signature genes across gastrointestinal tumours, single‐cell RNA sequencing (scRNA‐seq) datasets were obtained from six independent public cohorts of CRC and gastric cancer (GC), including datasets from the following published studies: Zhang et al. (CRC, GSE108989),^34^ Wu et al. (CRC liver metastasis, GSE41568),^35^ Kumar et al. (GC, GSE183904),^36^ Zheng et al. (pan‐cancer, GSE156728),^37^ Liu et al. (CRC liver metastasis, GSE164522)^38^ and Wang et al. (CRC liver metastasis, GSE50760).^39^

For each dataset, raw count matrices were processed using the Seurat v4 pipeline (Satija Lab). Quality control filters were applied to retain cells with 200–5000 detected genes, fewer than 20% mitochondrial reads, and a library size within three median absolute deviations of the dataset median. After log‐normalisation, the top 2000 highly variable genes were selected using the variance‐stabilising transformation method. Principal component analysis (PCA) was performed for dimensional reduction, and the first 20–30 significant principal components were used for graph‐based clustering (shared nearest neighbour algorithm) and non‐linear dimensional reduction via uniform manifold approximation and projection (UMAP).

### 2.17 Statistical analysis

Statistical analyses were performed with GraphPad Prism 8. Unpaired two‐tailed Student's *t*‐tests were used for two‐group comparisons, and one‐way/two‐way analysis of variance (ANOVA) with Dunnett's/Tukey's post hoc tests were used for multiple groups. For in vivo experiments, n represents the number of mice per group (biological replicates). For in vitro experiments, n represents the number of independent experiments performed on separate days with different cell preparations. The sample size (n), statistical methods and *p*‐values are provided in the figure captions.

## 3 RESULTS

### 3.1 Chronic stress accelerates CRC progression in preclinical models

**FIGURE 1:**
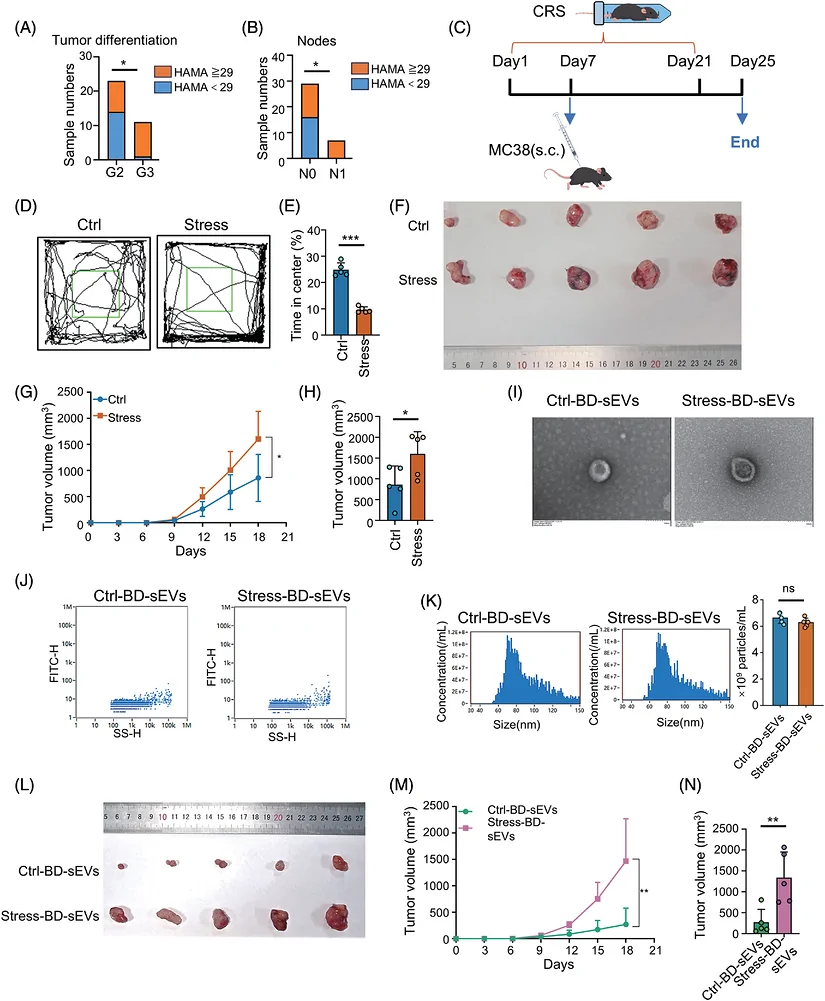
Effects of chronic stress and brain‐derived small extracellular vesicles (BD‐sEVs) on MC38 colorectal cancer (CRC) progression. (A and B) Correlation between Hamilton Anxiety Rating Scale (HAMA) scores and (A) tumour differentiation grade or (B) lymph node invasion in CRC patients (*n* = 37). (C) Schematic timeline of the MC38 subcutaneous model under chronic restraint stress (CRS). CRS was initiated on day 1; open field tests (OFT) were performed at day 7 (morning, prior to afternoon tumour inoculation), day 14 and day 21; MC38 cells (1 × 10^6^) were inoculated subcutaneously on day 7; CRS concluded at day 21; and endpoint at day 24. (D and E) OFT was performed on day 21 to evaluate anxiety‐like behaviours in C57BL/6 mice subjected to CRS or control conditions. Representative movement trajectories (D) and the percentage of time spent in the central zone (E) are shown (*n* = 5 mice per group). (F–H) Tumour growth in the MC38 subcutaneous model. Representative images of subcutaneous tumours (F), tumour volume growth curves (G) and final tumour volume at sacrifice on day 24 (H) are shown (*n* = 5 mice per group). (I–K) Characterisation of BD‐sEVs isolated from mouse plasma. (I) Transmission electron microscopy (TEM) image showing the typical cup‐shaped morphology (scale bar = 100 nm). (J) Flow cytometry detection of small extracellular vesicle (sEV) markers. (K) Nanoparticle tracking analysis (NTA) quantification of L1CAM^+^ BD‐sEV particle concentration in plasma from control (Ctrl) and CRS‐exposed (Stress) mice, showing no significant difference between groups (*n* = 4 mice per group). (L–N) Functional validation of BD‐sEV infusion. Starting on day 12, control BD‐sEVs (Ctrl‐BD‐sEVs) or stress‐induced BD‐sEVs (Stress‐BD‐sEVs) were injected into MC38 tumour‐bearing recipient mice via the tail vein every 3 days. Representative images of subcutaneous tumours (L), tumour volume growth curves (M) and final tumour volume at sacrifice on day 24 (N) are shown (*n* = 5 mice per group). Data are presented as mean ± SEM. ^*^ *p* < .05, ^**^ *p* < .01, ^***^ *p* < .001; n.s., not significant. Pearson's chi‐square test was used in (A) and (B). Unpaired two‐tailed Student's *t*‐test was used in (E), (H) and (K). Two‐way analysis of variance (ANOVA) was used in (G) and (M).

To explore the clinical relevance of chronic stress in CRC, we assessed anxiety in 37 CRC patients using the HAMA scale. The results revealed significant positive correlations between anxiety levels and tumour differentiation grade or lymph node invasion (Figure 1A,B), suggesting an association between anxiety and more advanced pathological features, including higher tumour grade and nodal involvement.

To validate this clinical observation, we established a CRS model in C57BL/6 mice. In this experimental design, stress was established before tumour challenge (CRS initiated on day 1; MC38 inoculation on day 7) and maintained throughout the tumour growth period (Figure 1C). OFT (Figures 1D–E and S1A–F) and sucrose preference tests (SPTs) (Figure S1G) confirmed the successful induction of anxiety‐like behaviours. Serial OFT revealed that stress‐induced behavioural changes emerged by day 14 and reached significance at day 21 (Figure S1A–F), suggesting that stress primarily acts on established tumours rather than exclusively facilitating initial engraftment. After subcutaneous inoculation of MC38 colon cancer cells, stressed mice exhibited significantly faster tumour growth and larger final tumour volumes than control mice did, confirming that stress accelerates CRC progression (Figure 1F–H).

To validate the generalisability of the Stress‐BD‐sEV–Treg axis beyond the subcutaneous MC38 model, we established an orthotopic CT26 CRC model in BALB/c mice using the same CRS paradigm (Figure S2A). Chronic stress significantly accelerated orthotopic tumour growth, as evidenced by increased tumour weight and volume compared with non‐stressed controls (Figure S2B,C). Flow cytometry analysis of orthotopic tumours confirmed elevated proportions of intratumoural FOXP3^+^ Treg in stressed mice (Figure S2D), mirroring the findings from the MC38 subcutaneous model. Serial OFT revealed an identical temporal pattern in the CT26 orthotopic model (Figure S2E–G). These data establish that the Stress‐BD‐sEV–Treg axis is reproducible across distinct CRC models.

### 3.2 BD‐sEVs mediate stress‐induced tumour acceleration

Immunostaining for c‐FOS (a marker of CNS activation) revealed robust activation of the hippocampal CA1–CA3 regions and the CPu in stressed mice (Figure S4A), consistent with stress‐induced neuronal activation across emotion‐regulating brain regions.^8^, ^12^, ^40^ To assess whether functional sEV secretion is restricted to these activated regions, we isolated sEVs from CA1–CA3, CPu, and other brain regions of stressed mice. sEVs from all three regions comparably activated STING signalling and promoted Treg differentiation in vitro, indicating that sEV secretion is a ubiquitous cellular process and that c‐FOS activation reflects regional stress responsiveness rather than exclusive sEV origin (Figure S4B–D).

We next evaluated the contribution of PNS‐sEVs to the circulating pool. PNS‐sEVs were enriched from mouse plasma using anti‐p75‐NTR antibodies in parallel with L1CAM‐based BD‐sEV enrichment from the same blood samples. Stress did not increase the protein mass of PNS‐sEVs in the circulation (Figure S4E,F). Functionally, Stress‐BD‐sEVs potently activated STING signalling and promoted Treg differentiation, whereas Stress‐PNS‐sEVs induced only marginal effects (Figure S4G–I), indicating that BD‐sEVs are the primary carriers of stress‐induced immunosuppressive signals.

To confirm that BD‐sEVs are key mediators in this process, we enriched L1CAM‐positive BD‐sEVs from mouse plasma and confirmed their sEV properties via TEM, flow cytometry and NTA (Figure 1I–K). Compared with Ctrl‐BD‐sEVs, intravenous injection of Stress‐BD‐sEVs significantly accelerated tumour growth in recipient mice (Figure 1L–N). These data identify BD‐sEVs as key components consistent with a role in stress–tumour crosstalk.

### 3.3 BD‐sEVs specifically promote the differentiation and immunosuppressive function of Treg in the TME

**FIGURE 2:**
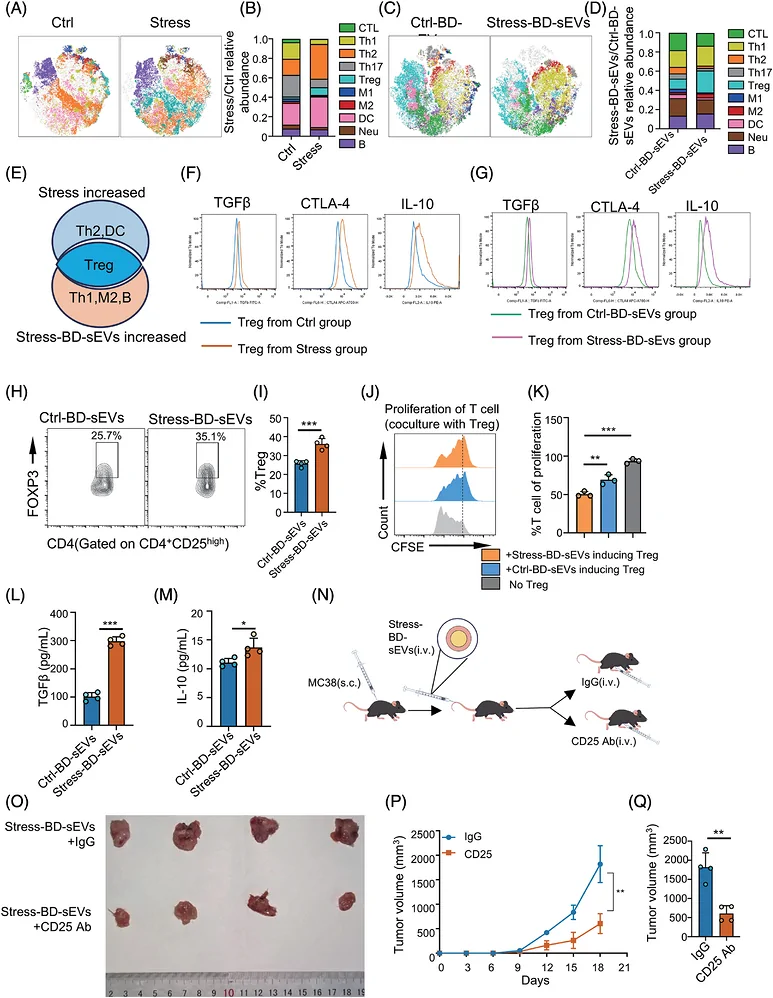
Effects of brain‐derived small extracellular vesicles (BD‐sEVs) on regulatory T (Treg) cell differentiation and immunosuppressive function in the tumour microenvironment. (A and B) C57BL/6 mice bearing MC38 subcutaneous tumours were subjected to chronic restraint stress (CRS) or control conditions. Tumour‐infiltrating immune cells were analysed by multicolour flow cytometry with t‐SNE dimensionality reduction (A), and the proportions of various immune cell subsets were quantified (B). Chronic stress predominantly increased tumour‐infiltrating FOXP3^+^ Treg, with other immune cell subsets (CD8^+^ T, CD4^+^FOXP3^−^, natural killer [NK], B, macrophage and dendritic cell [DC]) showing no significant changes (*n* = 5 mice per group). (C and D) MC38 tumour‐bearing mice were injected with control BD‐sEVs (Ctrl‐BD‐sEVs) or stress‐induced BD‐sEVs (Stress‐BD‐sEVs) via the tail vein every 3 days starting from day 5 after tumour inoculation. Tumour‐infiltrating immune cells were analysed by multicolour flow cytometry with t‐SNE dimensionality reduction (C), and the proportions of immune cell subsets were quantified (D) (*n* = 5 mice per group). (E) Venn diagram showing the intersection of immune cell subsets with significantly increased proportions in both the CRS model and the Stress‐BD‐sEV infusion model. (F and G) Tumour‐infiltrating Tregs were analysed for immunosuppressive marker expression. (F) In the CRS model, expression of cytotoxic T‐lymphocyte‐associated protein 4 (CTLA‐4), transforming growth factor‐β (TGF‐β) and interleukin‐10 (IL‐10) in tumour‐infiltrating Treg (*n* = 5 mice per group). (G) In the BD‐sEV infusion model, expression of CTLA‐4, TGF‐β and IL‐10 in tumour‐infiltrating Treg (*n* = 5 mice per group). (H and I) CD4^+^ naïve T cells isolated from the spleens of healthy C57BL/6 mice were treated with Ctrl‐BD‐sEVs or Stress‐BD‐sEVs for 72 h. Representative flow cytometry plots (H) and quantification of CD4^+^CD25^+^FOXP3^+^ Treg differentiation (I) are shown (*n* = 4 independent experiments). (J and K) Treg suppression assay. Treg preinduced by Ctrl‐BD‐sEVs or Stress‐BD‐sEVs were cocultured with CFSE‐labelled naïve CD4^+^ T cells. Representative flow cytometry plots (J) and quantification of the percentage of proliferating naïve T cells (K) are shown (*n* = 3 independent experiments). (L and M) CD4^+^ naïve T cells isolated from the spleens of healthy C57BL/6 mice were cocultured with Ctrl‐BD‐sEVs or Stress‐BD‐sEVs for 72 h. The concentrations of TGF‐β (L) and IL‐10 (M) in culture supernatants were measured by enzyme‐linked immunosorbent assay (ELISA) (*n* = 4 independent experiments). (N–Q) In vivo functional validation of Treg depletion. MC38 tumour‐bearing mice were intraperitoneally injected with anti‐CD25 depletion antibody or isotype control immunoglobulin G (IgG) along with Stress‐BD‐sEV infusion (N). Representative tumour images (O), tumour volume growth curves (P) and final tumour volume at sacrifice on day 24 (Q) are shown (*n* = 5 mice per group). Data are presented as mean ± SEM. ^*^ *p* < .05, ^**^ *p* < .01, ^***^ *p* < .001. Unpaired two‐tailed Student's *t*‐test was used in (B), (D), (F), (G), (I) and (K‒M). Two‐way analysis of variance (ANOVA) with Tukey's post hoc test was used in (P).

Having established the CNS origin and functional specificity of circulating BD‐sEVs, we next investigated their effects on the tumour immune microenvironment. To elucidate the mechanisms underlying the tumour‐promoting effects of Stress‐BD‐sEVs, we performed multicolour flow cytometry with t‐distributed stochastic neighbour embedding (t‐SNE) analysis of tumour‐infiltrating immune cells. Stress‐BD‐sEV treatment increased proportions of dendritic cells, T helper 2 (Th2) cells and Treg in the tumours of stressed mice (Figure 2A,B). Similarly, compared with the Ctrl‐BD‐sEV group, the Stress‐BD‐sEV group had significantly more tumour‐infiltrating Th1 cells, B cells, M2 macrophages and Treg (Figure 2C,D). Venn diagram analysis revealed Treg as the sole immune subset that was consistently elevated in both models (Figure 2E).

Functional analyses revealed that Treg from the chronic stress group or Stress‐BD‐sEV‐treated mice expressed more CTLA‐4 and secreted more TGF‐β and IL‐10, indicating enhanced immunosuppressive activity (Figure 2F,G). In vitro differentiation assays confirmed that Stress‐BD‐sEVs significantly promoted the differentiation of CD4^+^ naïve T cells into CD4^+^CD25^+^FOXP3^+^ Treg (Figure 2H,I), and these induced Treg suppressed naïve T‐cell proliferation more effectively (Figure 2J,K) and secreted more TGF‐β and IL‐10 (Figure 2L,M). Stress‐BD‐sEV‐induced Treg differentiation slightly increased in a dose‐dependent manner (Figure S6A,B).

To confirm that Treg are required for BD‐sEV‐mediated CRC progression, we depleted Treg in Stress‐BD‐sEV‐infused tumour‐bearing mice using anti‐CD25 antibodies (Figure 2N). Anti‐CD25 antibody treatment significantly reduced tumour volume (Figure 2O–Q), confirming that BD‐sEVs promote CRC progression via Treg‐mediated immunosuppression. Consistently, Treg depletion with anti‐CD25 antibodies in the direct CRS model also attenuated tumour growth (Figure S7), providing convergent evidence that Treg are essential for stress‐accelerated CRC progression across both experimental paradigms.

To determine whether BD‐sEV internalisation is required for Treg differentiation, we treated CD4^+^ naïve T cells with Stress‐BD‐sEVs in the presence of a sEV uptake inhibitor (heparin). The inhibitor significantly reduced Treg differentiation (Figure S6C), confirming that BD‐sEV uptake by CD4^+^ T cells is necessary for Treg induction.

To further characterise the cell‐type specificity of BD‐sEV effects, we isolated spleen‐derived CD4^+^ T cells, CD8^+^ T cells and bone marrow‐derived macrophages and treated each population with Ctrl‐BD‐sEVs or Stress‐BD‐sEVs. Among CD4^+^ T cells, Stress‐BD‐sEV treatment significantly increased the proportion of CD4^+^CD25^+^FOXP3^+^ Treg, whereas no significant differences were observed in IL‐4^+^ Th2 or IL‐17^+^ Th17 populations (Figure S6F,G), indicating that Stress‐BD‐sEVs specifically drive CD4^+^ T‐cell differentiation towards the Treg lineage rather than broadly activating effector T helper programs. In CD8^+^ T cells, Stress‐BD‐sEVs significantly upregulated IFN‐γ and Granzyme B expression, as well as the exhaustion marker T‐cell immunoglobulin and mucin‐domain containing‐3 (TIM‐3), while PD‐1 expression remained unchanged (Figure S6H,I), suggesting partial activation of effector and exhaustion programs. For bone marrow‐derived macrophages, the M2 activation marker (CD80^−^CD206^+^) showed no significant difference between groups, whereas the M1 marker (CD80^+^MHC‐II^+^) was modestly but significantly elevated in the Stress‐BD‐sEV group (Figure S6J,K). These data confirm that although Stress‐BD‐sEVs exert partial effects on CD8^+^ T‐cell activation and macrophage M1 polarisation, CD4^+^ T cells—and specifically Treg differentiation—represent the primary and most robust functional target of Stress‐BD‐sEVs.

As controls, we confirmed that Stress‐BD‐sEVs had no direct effects on CRC cell viability or apoptosis (Figure S5A–C), and did not upregulate NOXA or activate STING signalling in MC38 or CT26 cells (Figure S5D–F). These data suggest that the Stress‐BD‐sEV‒Treg axis may influence immune checkpoint profiles.

### 3.4 Plasma L1CAM‐enriched sEVs exhibit multi‐dimensional equivalence to brain‐tissue‐derived sEVs

Although anti‐L1CAM antibody‐based BD‐sEV isolation is a widely used approach, concerns about nonneural contamination persist.^11^ As baseline characterisation, brain‐tissue sEVs (brain‐sEVs) isolated directly from mouse brain tissue exhibited canonical sEV morphology by TEM, confirmed by flow cytometry, and showed a size distribution of 30–150 nm by NTA (Figure S11A–C). At the protein level, BD‐sEVs and directly isolated brain‐sEVs shared neuronal markers (synaptophysin, NeuN) and canonical EV markers (CD63 and TSG101), with minimal glial fibrillary acidic protein (GFAP) contamination in both (Figure S11K). At the miRNA level, BD‐sEVs and brain‐sEVs displayed highly correlated expression profiles (Figure S12A). The miRNA‐seq revealed that brain‐enriched miRNAs (miR‐124‐3p, miR‐9‐5p and miR‐128‐1‐5p) were detected at high levels in both BD‐sEVs and brain‐sEVs (Figure S12B). Gene Ontology (GO) enrichment analysis of brain‐sEV miRNA targets revealed significant overlap with BD‐sEV target profiles, and Venn diagram comparison confirmed convergent GO terms between brain‐sEVs and circulating BD‐sEVs (Figure S11E–J). Functionally, L1CAM‐depleted sEVs lost their Treg‐inducing capacity (Figure S13A–F), and stress brain‐sEVs recapitulated the complete functional signature of Stress‐BD‐sEVs—Treg differentiation, NOXA upregulation, STING activation and mtDNA release—at comparable magnitudes (Figure S13G–K). Together, these multi‐dimensional data provide convergent evidence that the functional activity of circulating L1CAM^+^ BD‐sEVs reflects a CNS origin.

### 3.5 BD‐sEVs promote Treg differentiation by upregulating NOXA expression in CD4^+^ naïve T cells

**FIGURE 3:**
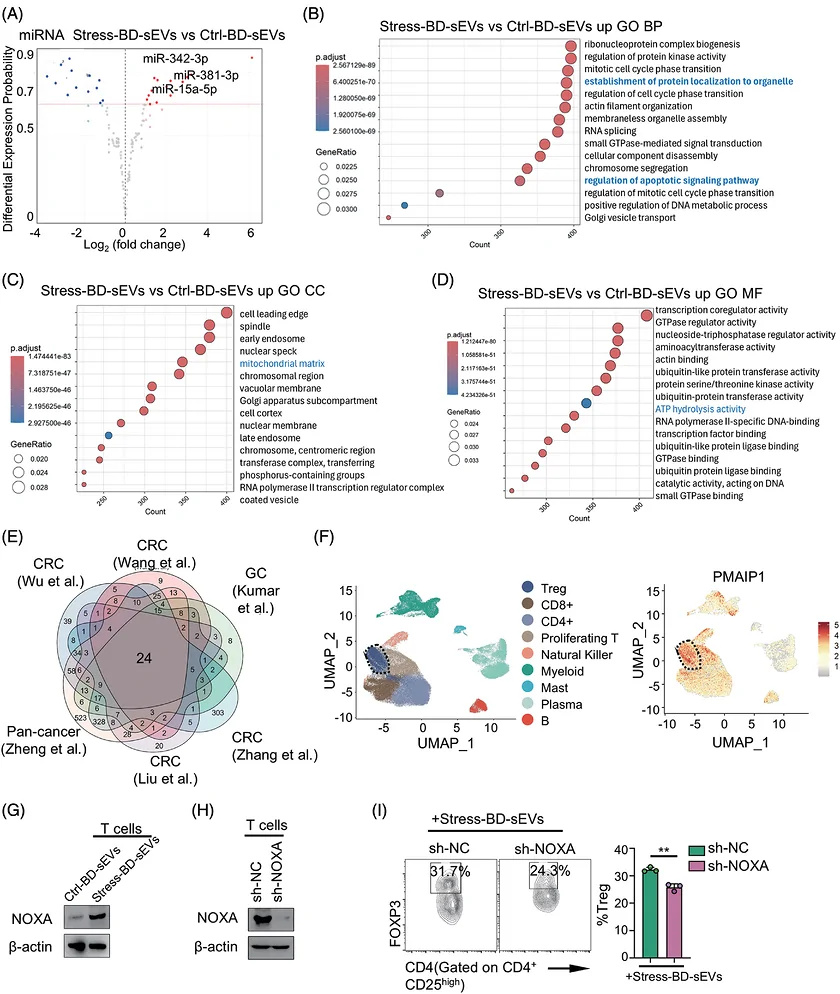
Role of phorbol‐12‐myristate‐13‐acetate‐induced protein 1 (NOXA) in brain‐derived small extracellular vesicle (BD‐sEV)‐mediated regulatory T cell (Treg) differentiation in CD4^+^ naïve T cells. (A) Volcano plot showing differentially expressed microRNAs (miRNAs) in stress‐induced BD‐sEVs (Stress‐BD‐sEVs) compared with control BD‐sEVs (Ctrl‐BD‐sEVs). (B–D) Gene Ontology (GO) enrichment analysis of target genes of differentially expressed miRNAs in BD‐sEVs: (B) biological process (BP); (C) cellular component (CC); and (D) molecular function (MF). (E) Venn diagram showing the intersection of 24 genes highly expressed in tumour‐infiltrating Treg across six public single‐cell RNA sequencing (scRNA‐seq) datasets of gastrointestinal tumours, including colorectal cancer (CRC) and gastric cancer (GC). (F) Reanalysis of a published CRC scRNA‐seq dataset. Uniform manifold approximation and projection (UMAP) dimensionality reduction plot of tumour‐infiltrating lymphocyte (TIL) subsets (left); heatmap showing the transcription level of the *PMAIP1* gene (encoding the NOXA protein) in each TIL subset (right). (G) CD4^+^ naïve T cells isolated from the spleens of healthy C57BL/6 mice were treated with Ctrl‐BD‐sEVs or Stress‐BD‐sEVs for 72 h. NOXA protein expression was detected by Western blotting (*n* = 3 independent experiments). (H and I) CD4^+^ naïve T cells were infected with lentivirus expressing sh‐NOXA (NOXA knockdown) or sh‐NC (negative control). Western blotting was performed to confirm knockdown efficiency (H). Cells were treated with Stress‐BD‐sEVs for 72 h, and the differentiation ratio of CD4^+^CD25^+^FOXP3^+^ Treg was determined by flow cytometry (I) (*n* = 3 independent experiments). Data are presented as mean ± SEM. ^*^ *p* < .05, ^**^ *p* < .01, ^***^ *p* < .001. Unpaired two‐tailed Student's *t*‐test was used in (G) and (I).

To identify the specific miRNA cargo that may regulate NOXA expression in recipient T cells, we performed miRNA sequencing on Ctrl‐BD‐sEVs and Stress‐BD‐sEVs and revealed significant upregulation of miR‐342‐3p, miR‐15a‐5p and miR‐381‐3p in Stress‐BD‐sEVs (Figure 3A). Previous research revealed that these miRNAs regulate T‐cell differentiation, mitochondrial function and IFN‐I pathways: miR‐342‐3p targets Rictor to modulate Treg function/mitochondrial homeostasis; miR‐15a‐5p affects T‐cell activation via the phosphatase and tensin homolog–protein kinase B (PTEN‐AKT) axis and drives IFN‐I production by targeting MCL1; and miR‐381‐3p has been reported to upregulate NOXA expression, potentially through MDM2 inhibition.^41^, ^42^, ^43^, ^44^ Quantitative reverse transcription‐polymerase chain reaction (qRT‐PCR) validation confirmed the upregulation of miR‐342‐3p, miR‐15a‐5p and miR‐381‐3p in Stress‐BD‐sEVs (Figure S8A). GO enrichment analysis of target genes revealed significant enrichment in the mitochondrial matrix, apoptotic signalling and protein subcellular localisation, suggesting that these miRNAs regulate T‐cell differentiation by altering mitochondrial and apoptotic pathways in recipient cells (Figure 3B–D). Construction of a ceRNA network centred on these stress‐responsive miRNAs and Kyoto Encyclopedia of Genes and Genomes (KEGG) pathway enrichment analysis further supported their involvement in PI3K‒Akt signalling and apoptosis regulation (Figure S12C,D).

Integration of scRNA‐seq data from six gastrointestinal cancer datasets revealed 24 conserved genes highly expressed in Treg, including PMAIP1 (encoding NOXA) and FOXP3 (a Treg marker) (Figures 3E and S10A). UMAP analysis confirmed that PMAIP1 was specifically enriched in CRC Treg clusters (Figures 3F and S10C), with the highest expression in functionally potent Fr.II effector Treg (Figure S10B). PMAIP1 was also specifically upregulated in Treg across multiple malignancies (non‐small cell lung cancer, liver cancer, breast cancer, glioma and endometrial cancer), confirming its conserved role in Treg (Figure S10D–L).

To determine whether the enhanced Treg‐inducing capacity of Stress‐BD‐sEVs is driven by changes in particle quantity or RNA cargo composition, we first performed NTA quantification of L1CAM^+^ BD‐sEVs, which revealed no significant difference in particle concentration between Ctrl and Stress mice (Figure 1K), ruling out increased secretion as the source of functional divergence. To directly dissect the contribution of cargo molecules, Stress‐BD‐sEVs were pre‐treated with protease K or RNase A and applied to CD4^+^ naïve T cells at equal input doses. Stress‐BD‐sEVs remained significantly more potent than Ctrl‐BD‐sEVs even after protease K treatment, whereas RNase pre‐treatment largely abrogated the enhanced Treg‐inducing capacity and NOXA upregulation (Figure S8B,C). These data indicate that the RNA component, rather than the protein component, is the primary molecular driver of stress‐enhanced immunosuppressive activity.

Consistent with the bioinformatic predictions, both Ctrl‐BD‐sEVs and Stress‐BD‐sEVs upregulated NOXA protein expression in CD4^+^ naïve T cells, with Stress‐BD‐sEVs having a stronger effect (Figure 3G). Compared with the control (sh‐NC), NOXA knockdown (via sh‐NOXA) significantly attenuated Stress‐BD‐sEV‐induced Treg differentiation (Figure 3H,I).

To directly test whether the three upregulated miRNAs are sufficient to recapitulate the effects of Stress‐BD‐sEVs, we performed gain‐of‐function experiments using synthetic miRNA mimics. CD4^+^ naïve T cells were electroporated with mimic NC, miR‐342‐3p mimic, miR‐381‐3p mimic or miR‐15a‐5p mimic under Ctrl‐sEV co‐treatment conditions, with a Stress‐BD‐sEV + mimic‐NC group as positive control. Among the three miRNAs, miR‐342‐3p showed the strongest effect, significantly upregulating NOXA protein levels and increasing Treg differentiation compared with the mimic NC control, whereas miR‐381‐3p and miR‐15a‐5p each induced partial but detectable effects (Figure S8D–E). No single miRNA mimic fully recapitulated the magnitude of Stress‐sEV‐induced Treg differentiation, qRT‐PCR confirmed successful overexpression of each miRNA after electroporation (Figure S8F). These data indicate that the complete phenotype likely requires synergistic contributions from multiple miRNAs.

Complementary loss‐of‐function experiments using antagomirs further confirmed the necessity of these miRNAs. CD4^+^ naïve T cells were electroporated with antagomir‐NC, antagomir‐miR‐342‐3p, antagomir‐miR‐381‐3p or antagomir‐miR‐15a‐5p, followed by Stress‐BD‐sEV treatment. All the three antagomir‐miRNA produced the attenuation of NOXA protein levels and Treg differentiation (Figure S9A–C). Annexin V/PI analysis confirmed that antagomir treatment did not affect cell viability (Figure S9D), ruling out non‐specific cytotoxicity. No single antagomir completely abolished the Stress‐BD‐sEV phenotype, supporting a cooperative multi‐miRNA model in which miR‐342‐3p provides the dominant contribution while miR‐381‐3p and miR‐15a‐5p supply complementary inputs. Bidirectional genetic evidence that miRNA mimics partially induce the phenotype and antagomirs partially suppress it supports a cooperative multi‐miRNA model in which miR‐342‐3p, miR‐381‐3p and miR‐15a‐5p delivered by Stress‐BD‐sEVs contribute to NOXA upregulation and subsequent Treg differentiation. However, we note that residual Treg differentiation after single antagomir treatment may also reflect contributions from non‐miRNA cargo (protein, lipid or other RNA species), and the complete functional output of the Stress‐BD‐sEV phenotype likely involves both miRNA synergy and non‐miRNA components.

To demonstrate the operation of the BD‐sEV–miRNA–NOXA axis in vivo under conditions of concomitant HPA and SNS activation, we performed a three‐arm CT26 orthotopic CRC model with pharmacological HPA/SNS blockade. CT26 tumour‐bearing BALB/c mice were assigned to Ctrl, CRS (Stress), and Stress plus propranolol + mifepristone (Stress + PROP + RU‐486) groups. BD‐sEVs isolated from plasma of Stress + PROP + RU‐486 mice showed significantly attenuated upregulation of miR‐342‐3p, miR‐15a‐5p and miR‐381‐3p compared with Stress BD‐sEVs (Figures S2A,B and S3A). In functional transfer assays, BD‐sEVs from Stress mice potently promoted Treg differentiation and upregulated NOXA protein in naive CD4^+^ T cells, whereas BD‐sEVs from Stress + PROP + RU‐486 mice showed significantly impaired Treg‐inducing capacity and attenuated NOXA induction (Figure S3B–E). Because the drug treatments were never applied directly to the in vitro culture, the functional deficit must reside in the BD‐sEV cargo itself. Intratumoural Treg from Stress mice showed the highest NOXA protein expression and STING pathway activation (p‐TBK1 and p‐IRF3), which were partially attenuated in Stress + PROP + RU‐486 Treg (Figure S3F,G). These data provide in vivo evidence that the BD‐sEV–miRNA–NOXA–STING axis is regulated by upstream HPA/SNS signalling.

### 3.6 NOXA binds HSP60 to induce mitochondrial dysfunction and non‐apoptotic mtDNA release

**FIGURE 4:**
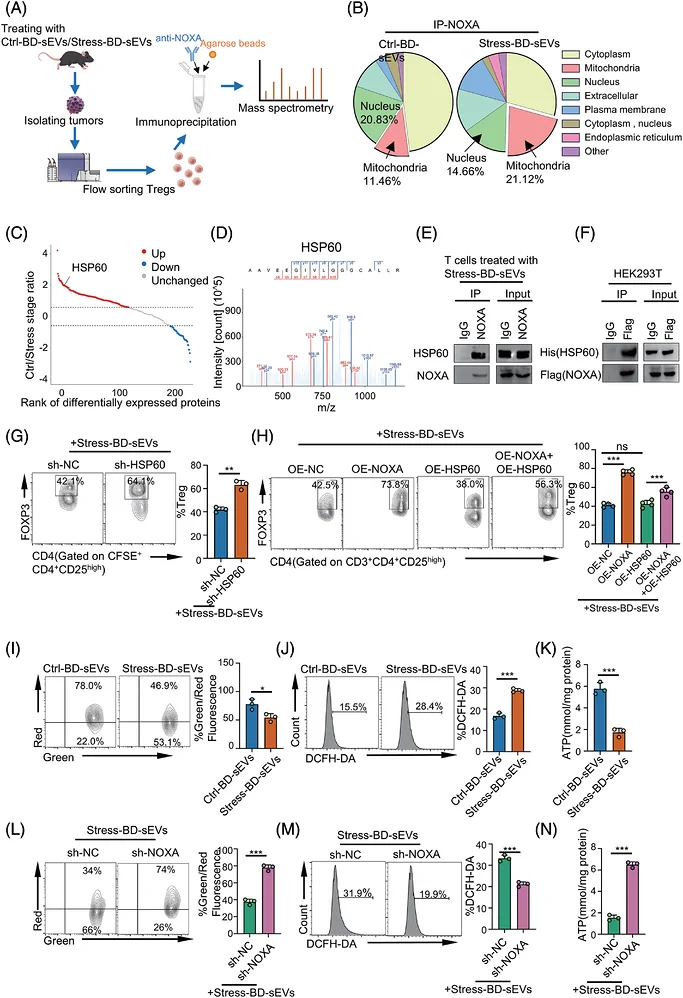
Phorbol‐12‐myristate‐13‐acetate‐induced protein 1 (NOXA)‒heat shock protein 60 (HSP60) interaction and mitochondrial function during brain‐derived small extracellular vesicle (BD‐sEV)‐mediated regulatory T cell (Treg) differentiation. (A) Schematic of the workflow for identifying NOXA‐interacting proteins. C57BL/6 mice bearing subcutaneous tumours were treated with control BD‐sEVs (Ctrl‐BD‐sEVs) or stress‐induced BD‐sEVs (Stress‐BD‐sEVs). Tregs from the tumour microenvironment were enriched by flow cytometry sorting, followed by co‐immunoprecipitation (Co‐IP) using an anti‐NOXA antibody and mass spectrometry (MS)‐based proteomic analysis. (B) MS results revealing that NOXA‐interacting components in the Stress‐BD‐sEV‐treated group were significantly enriched in mitochondria‐related proteins compared with the Ctrl‐BD‐sEV group. (C) MS analysis of NOXA‐binding proteins showing that HSP60 (*HSPD1*), a key mitochondria‐related molecule, was among the top 10 interacting proteins. (D) Representative MS spectrum of HSP60 peptides in the NOXA Co‐IP complex. (E) Endogenous Co‐IP was used to detect the interaction between endogenous NOXA and HSP60 in CD4^+^ naïve T cells isolated from the spleens of healthy C57BL/6 mice and treated with Ctrl‐BD‐sEVs or Stress‐BD‐sEVs for 72 h. Stress‐BD‐sEV treatment enhanced NOXA‒HSP60 binding. (F) Validation of the interaction in a coexpression system. 293T cells were cotransfected with Flag‐tagged NOXA and His‐tagged HSP60. After Co‐IP using an anti‐Flag antibody, immunoblotting with an anti‐His antibody confirmed direct binding. (G) CD4^+^ naïve T cells infected with lentivirus expressing sh‐HSP60 or sh‐NC were treated with Stress‐BD‐sEVs for 72 h. Representative flow cytometry plots and quantification of CD4^+^CD25^+^FOXP3^+^ Treg differentiation are shown. HSP60 knockdown significantly increased Treg differentiation, consistent with the model in which HSP60 loss phenocopies NOXA‐mediated inhibition of HSP60 function (*n* = 3 independent experiments). (H) CD4^+^ naïve T cells were infected with overexpression lentiviruses (OE‐NC, OE‐NOXA, OE‐HSP60, or OE‐NOXA + OE‐HSP60). After treatment with Stress‐BD‐sEVs for 72 h, the Treg differentiation ratio was determined by flow cytometry. Representative flow cytometry plots and quantification from four independent replicates are shown (*n* = 4 independent experiments). (I–K) Detection of mitochondrial function in CD4^+^ naïve T cells treated with Ctrl‐BD‐sEVs or Stress‐BD‐sEVs for 72 h. Mitochondrial membrane potential measured by JC‐1 staining (I); intracellular reactive oxygen species (ROS) levels detected by DCFH‐DA fluorescent probe (J); and adenosine triphosphate (ATP) production measured by chemiluminescence (K) (*n* = 3 independent experiments). (L–N) CD4^+^ naïve T cells with lentiviral‐mediated short hairpin RNA (shRNA) NOXA knockdown (sh‐NOXA) or negative control shRNA (sh‐NC) were treated with Stress‐BD‐sEVs for 72 h. Mitochondrial membrane potential (L), ROS levels (M) and ATP production (N) were detected (*n* = 3 independent experiments). Data are presented as mean ± SEM. ^*^ *p* < .05, ^**^ *p* < .01, ^***^ *p* < .001. Unpaired two‐tailed Student's *t*‐test was used in (E), (G), (I–K) and (L–N). One‐way analysis of variance (ANOVA) with Tukey's post hoc test was used in (H).

To explore the downstream mechanisms of NOXA, we performed anti‐NOXA co‐immunoprecipitation (Co‐IP) in Stress‐BD‐sEV‐treated CD4^+^ naïve T cells, followed by MS (Figure 4A). Mitochondrial proteins were enriched in NOXA‐interacting complexes (Figures 4B and S14A,B), highlighting the role of mitochondrial function in Treg differentiation.

The mitochondrial chaperone HSP60 (HSPD1) was identified as a key interactor, as it ranked among the top ten most abundant proteins according to the MS data, and its function is most closely linked to that of both the miRNAs in Stress‐BD‐sEVs and NOXA activity (Figures 4C,D and S14C). Co‐IP confirmed the endogenous NOXA–HSP60 interaction in Stress‐BD‐sEV‐treated CD4^+^ naïve T cells (Figure 4E). The interaction between NOXA and HSP60 was also confirmed in 293T cells with exogenous expression (Figure 4F). Functionally, HSP60 knockdown increased Stress‐sEV‐induced Treg differentiation (Figures 4G and S14D), consistent with the model in which HSP60 loss phenocopies NOXA‐mediated inhibition of HSP60 function. NOXA overexpression promoted Treg generation, whereas co‐overexpression of HSP60 antagonised this effect (Figures 4H and S14E). Together, these data indicate that NOXA inhibits the function of HSP60 through direct binding and chaperone activity suppression. Notably, HSP60 total protein levels were unchanged by Stress‐sEV treatment, indicating that the functional effect is mediated through the NOXA–HSP60 interaction rather than through altered HSP60 expression (Figure S11D).

Functional analysis revealed that Stress‐BD‐sEV treatment reduced the mitochondrial membrane potential of CD4^+^ naïve T cells, increased ROS production and decreased ATP generation (Figure 4I–K), whereas NOXA knockdown reversed the mitochondrial dysfunction induced by Stress‐BD‐sEVs (Figure 4L–N). These data indicate that NOXA is required for Stress‐sEV‐induced mitochondrial dysfunction in T cells.

To distinguish the observed mitochondrial dysfunction from classical apoptotic signalling, we performed digitonin‐based subcellular fractionation. Stress‐BD‐sEV treatment significantly elevated cytosolic mtDNA (ND1/ND5) whereas cytosolic cytochrome c remained at background levels and the mitochondrial marker COX IV‐ was low expression confined to the cytosolic fraction (Figure S15A,B), indicating selective inner‐membrane permeabilisation rather than BAX/BAK‐driven mitochondrial outer membrane permeabilization (MOMP). Concurrent analysis confirmed that CD4^+^ naïve T cells remained >90% viable after Stress‐BD‐sEV treatment, with minimal CellEvent Caspase‐3/7 activation (Figure S15C,E). Annexin V binding partly reflected activation‐induced phosphatidylserine externalisation rather than apoptotic execution, and shRNA‐mediated NOXA knockdown attenuated both the Annexin V signal and Treg differentiation (Figure S15C,D). These data are consistent with a model in which NOXA signalling functionally diverges downstream of MCL‐1: the canonical pro‐apoptotic arm is MCL‐1 dependent, whereas the mtDNA‐release/Treg‐polarising arm operates through HSP60 inhibition. We note that formal proof of pathway bifurcation would require domain‐specific NOXA mutants that selectively disrupt MCL‐1 binding while preserving HSP60 interaction.

To determine whether Treg differentiation is a downstream consequence of NOXA's canonical MCL‐1 sequestration, we overexpressed MCL‐1 in CD4^+^ naïve T cells under OE‐NOXA conditions. MCL‐1 overexpression significantly attenuated Annexin V^+^ cells (Figure S15G), confirming rescue of the pro‐apoptotic branch. Critically, MCL‐1 overexpression did not rescue cytosolic mtDNA release, STING pathway activation (p‐TBK1/p‐IRF3) or Treg differentiation (Figure S15F,H–J). This experiment genetically separates NOXA's downstream effects into an MCL‐1‐dependent pro‐apoptotic branch and an MCL‐1‐independent, HSP60‐dependent Treg‐polarising branch.

To determine the biochemical mechanism of NOXA‐mediated HSP60 negative regulation, we performed three sets of experiments. Cycloheximide (CHX) chase assays revealed that HSP60 protein levels remained stable over 48 h in both OE‐NC and OE‐NOXA groups, with no detectable difference in decay rates (Figure S16A). HSP60 ubiquitination levels were comparable between OE‐NC and OE‐NOXA groups (Figure S16B), confirming that NOXA does not promote proteasomal degradation. Using a citrate synthase (CS) thermal aggregation assay, IP‐HSP60 from OE‐NOXA cells showed significantly reduced suppression of CS aggregation compared with IP‐HSP60 from OE‐NC cells (Figure S16C), demonstrating that NOXA binding directly impairs HSP60 chaperone activity. Together, these data establish that NOXA negatively regulates HSP60 by direct binding and inhibition of its chaperone function, without promoting degradation or blocking mitochondrial import.

To directly test whether the observed mtDNA release is driven by alternative stress pathways, we treated Stress‐BD‐sEV‐stimulated CD4^+^ naïve T cells with pathway‐specific inhibitors: NIM811 (mPTP inhibitor), VBIT‐4 (VDAC oligomerisation inhibitor), BAI1 (BAX channel inhibitor) and NAC (ROS scavenger). NIM811 and VBIT‐4 produced no significant attenuation of cytosolic mtDNA release, STING pathway activation, Treg differentiation, or IFN‐β secretion compared with the DMSO vehicle control, directly excluding mPTP opening and VDAC oligomerisation as operative mechanisms (Figure S17A–E). BAI1 and NAC each produced modest but statistically significant attenuation, suggesting their contributions are partial rather than primary driving mechanisms. Pharmacological exclusion experiments thus rule out mPTP, VDAC, BAX/MOMP and ROS as primary drivers, supporting a model in which the NOXA–HSP60 interaction is the specific and functionally dominant mechanism of mtDNA release in this system.

### 3.7 Cytosolic mtDNA activates cGAS‒STING‒IFN‐I to drive Treg differentiation

**FIGURE 5:**
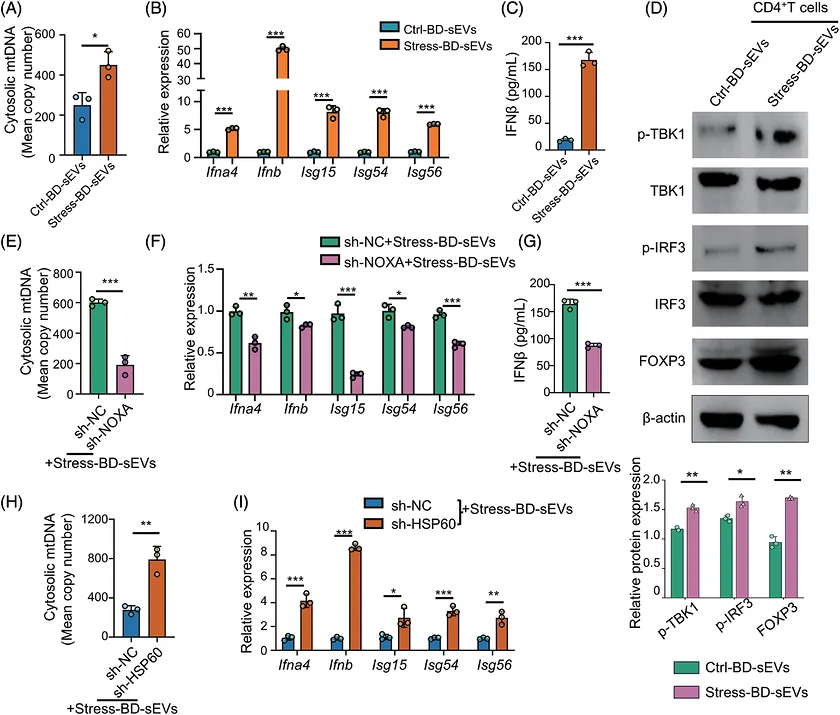
Effects of stress‐induced brain‐derived small extracellular vesicles (Stress‐BD‐sEVs) on cytosolic mitochondrial DNA (mtDNA) release and type I interferon (IFN‐I) pathway activation in CD4^+^ naïve T cells. (A–C) CD4^+^ naïve T cells isolated from the spleens of healthy C57BL/6 mice were treated with control BD‐sEVs (Ctrl‐BD‐sEVs) or Stress‐BD‐sEVs for 72 h. Cytoplasmic mtDNA levels were quantified by quantitative PCR (qPCR) (A). The mRNA expression of IFN‐I‐related genes was detected by qPCR (B). The IFN‐β concentration in cell culture supernatants was measured by enzyme‐linked immunosorbent assay (ELISA) (C) (*n* = 3 independent experiments). (D) Activation of the stimulator of interferon genes (STING) pathway was examined by Western blotting. CD4^+^ naïve T cells were treated with Ctrl‐BD‐sEVs or Stress‐BD‐sEVs (10 µg/mL) for 2 h, and p‐TBK1, TBK1, p‐IRF3, IRF3 and Forkhead box P3 (FOXP3) were detected with β‐actin as loading control. Three independent biological replicates are shown, with ImageJ grey‐scale quantification normalised to β‐actin (*n* = 3 independent experiments). (E–G) CD4^+^ naïve T cells infected with lentivirus expressing sh‐NOXA or sh‐NC were treated with Stress‐BD‐sEVs for 72 h. Cytoplasmic mtDNA levels were quantified by qPCR (E). The mRNA expression of IFN‐I‐related genes was analysed by qPCR (F). The IFN‐β concentration in the supernatants was detected by ELISA (G) (*n* = 3 independent experiments). (H and I) CD4^+^ naïve T cells infected with lentivirus expressing sh‐HSP60 or sh‐NC were treated with Stress‐BD‐sEVs for 72 h. The mRNA expression of IFN‐I‐related genes was analysed by qPCR (H). The IFN‐β concentration in the supernatants was detected by ELISA (I) (*n* = 3 independent experiments). Data are presented as mean ± SEM. ^*^ *p* < .05, ^**^ *p* < .01, ^***^ *p* < .001. Unpaired two‐tailed Student's *t*‐test was used in (A–C), (E–G), (H) and (I).

Stress‐BD‐sEV treatment significantly increased cytoplasmic mtDNA levels in CD4^+^ naïve T cells, upregulated IFN‐I‐related gene expression, increased the concentration of IFN‐β in the supernatant, and activated the STING pathway (Figure 5A–D). NOXA knockdown reduced cytoplasmic mtDNA release, IFN‐I‐related gene expression and IFN‐β secretion in Stress‐BD‐sEV‐treated cells (Figure 5E–G). Conversely, HSP60 knockdown increased IFN‐I‐related gene expression and IFN‐β secretion (Figure 5H,I). These data confirm that mitochondrial dysfunction and mtDNA release mediated by NOXA‒HSP60 can activate the IFN‐I pathway.

### 3.8 NOXA and FOXP3 expression are positively correlated with patient anxiety levels and predict poor prognosis

**FIGURE 6:**
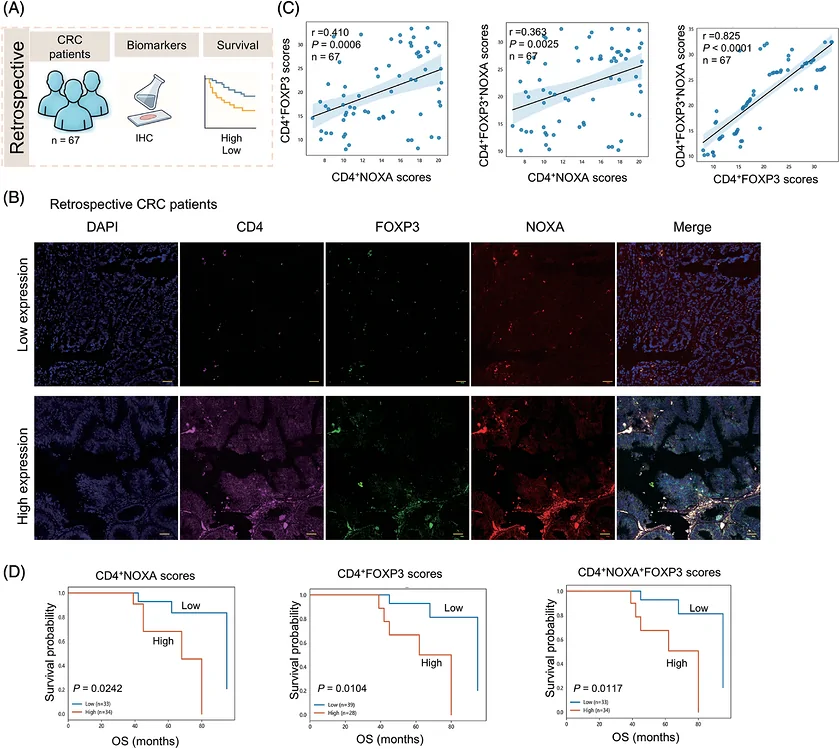
Intratumoural phorbol‐12‐myristate‐13‐acetate‐induced protein 1 (NOXA) and Forkhead box P3 (FOXP3) expression and prognosis in a retrospective colorectal cancer (CRC) patient cohort. (A) Flowchart of the retrospective cohort study involving 67 CRC patients. (B) Representative multicolour immunofluorescence images of CRC tumour tissues from the retrospective cohort. CD4 (magenta), FOXP3 (green), NOXA (red) and DAPI (blue) staining showing intracellular co‐localisation of NOXA and FOXP3 within CD4^+^ cells. Scale bar = 50 µm. (C) Pearson correlation analysis of CD4^+^NOXA^+^, CD4^+^FOXP3^+^ and CD4^+^FOXP3^+^NOXA^+^ cell densities in the retrospective cohort (*n* = 67). Three panels of pairwise scatter plots showing significant positive correlations among all three indices. (D) Kaplan‒Meier curves of overall survival (OS) of CRC patients stratified by CD4^+^NOXA^+^, CD4^+^FOXP3^+^ and CD4^+^FOXP3^+^NOXA^+^ cell densities (high vs. low). Three panels showing that high expression of all three indices was associated with poor prognosis. Differences between groups were assessed using the log‐rank test (*n* = 67). Data are presented as mean ± SEM. ^*^ *p* < .05, ^**^ *p* < .01, ^***^ *p* < .001. Pearson correlation analysis was used in (C). Log‐rank test was used in (D).

To determine the clinical relevance of our findings, we first conducted a retrospective analysis of 67 CRC patients (Figure 6A and Table S1, retrospective cohort, *n* = 67). Representative multicolour immunofluorescence confirmed intracellular co‐localisation of NOXA and FOXP3 within CD4^+^ cells in tumour tissues (Figure 6B). Pearson correlation analysis revealed significant positive correlations among CD4^+^NOXA^+^, CD4^+^FOXP3^+^ and CD4^+^FOXP3^+^NOXA^+^ cell densities (Figure 6C). Kaplan‒Meier analysis showed that high expression of all three indices was associated with poor overall survival (Figure 6D, retrospective cohort, *n* = 67).

**FIGURE 7:**
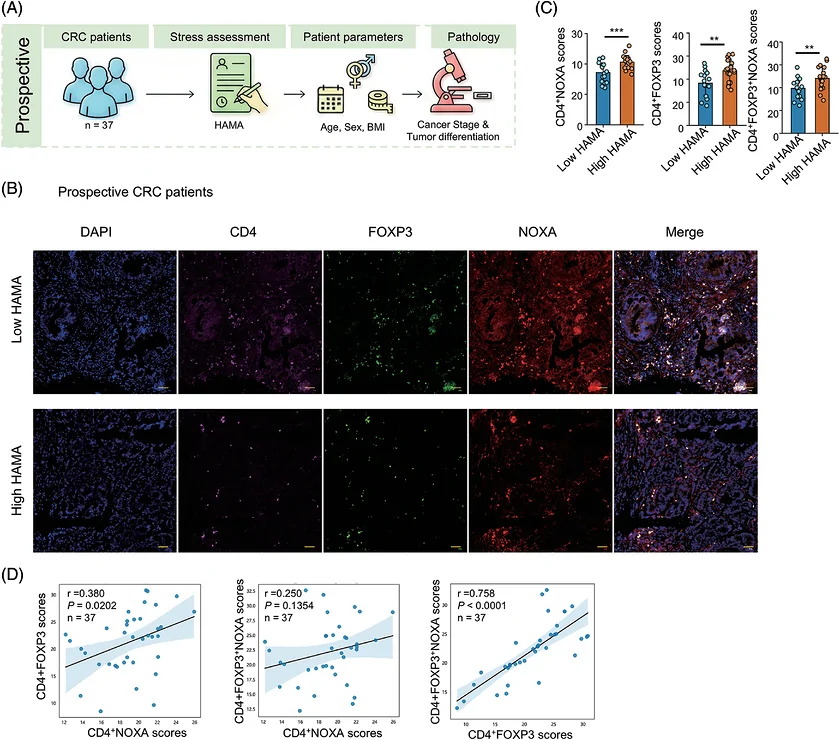
Intratumoural phorbol‐12‐myristate‐13‐acetate‐induced protein 1 (NOXA) and Forkhead box P3 (FOXP3) expression in relation to preoperative anxiety levels in a prospective colorectal cancer (CRC) patient cohort. (A) Schematic of the prospective cohort study of 37 CRC patients, including the assessment of anxiety levels using the Hamilton Anxiety Rating Scale (HAMA). (B) Representative multicolour immunofluorescence images of CRC tumour tissues from the prospective cohort, stratified by low and high HAMA scores. CD4 (magenta), FOXP3 (green), NOXA (red) and DAPI (blue) staining. Higher NOXA and FOXP3 signal intensities were observed in the high‐HAMA group. Scale bar = 50 µm. (C) Quantification of CD4^+^NOXA^+^, CD4^+^FOXP3^+^ and CD4^+^FOXP3^+^NOXA^+^ cell densities in low‐HAMA versus high‐HAMA groups (*n* = 37). All three indices were significantly higher in the high‐HAMA group. (D) Pearson correlation analysis of CD4^+^NOXA^+^, CD4^+^FOXP3^+^ and CD4^+^FOXP3^+^NOXA^+^ cell densities in the prospective cohort (*n* = 37). Three panels of pairwise scatter plots showing significant positive correlations between CD4^+^FOXP3^+^ and CD4^+^FOXP3^+^NOXA^+^, between CD4^+^FOXP3^+^ and CD4^+^NOXA^+^, and between CD4^+^NOXA^+^ and CD4^+^FOXP3^+^NOXA^+^. Data are presented as mean ± SEM. ^*^ *p* < .05, ^**^ *p* < .01, ^***^ *p* < .001. Unpaired two‐tailed Student's *t*‐test was used in (C). Pearson correlation analysis was used in (D).

We further validated these findings in a prospective cohort of 37 CRC patients with preoperative HAMA anxiety assessment (Figure 7A and Table S2, prospective cohort, *n* = 37). Multicolour immunofluorescence showed higher NOXA and FOXP3 signal intensities in the high‐HAMA group compared with the low‐HAMA group (Figure 7B). Quantification confirmed that CD4^+^NOXA^+^, CD4^+^FOXP3^+^ and CD4^+^FOXP3^+^NOXA^+^ cell densities were all significantly elevated in high‐HAMA patients (Figure 7C). Pearson correlation analysis revealed significant positive correlations between CD4^+^FOXP3^+^ Treg density and the other two indices (CD4^+^FOXP3^+^ vs. CD4^+^FOXP3^+^NOXA^+^, CD4^+^FOXP3^+^ vs. CD4^+^NOXA^+^) in this prospective cohort (Figure 7D).

To further characterise the tumour immune microenvironment in the context of preoperative anxiety, we performed multicolour immunofluorescence staining for DAPI, CD8, PD‐L1 and PD‐1 on tumour sections from CRC patients stratified by HAMA scores. High‐HAMA patients exhibited reduced CD8^+^ T‐cell density, reduced PD‐L1 expression and reduced PD‐1^+^ cell proportion compared with low‐HAMA patients (Figure S18A,B), indicating an immunosuppressive and immune‐exhausted TME associated with elevated anxiety.

Correlation analysis of clinicopathological characteristics revealed that in the retrospective cohort, high CD4^+^FOXP3^+^ and CD4^+^FOXP3^+^NOXA^+^ expression were each significantly associated with poorer tumour differentiation (*p* = .0128 and .002, respectively). In the prospective cohort, high preoperative HAMA scores were significantly associated with multiple adverse features, including poorer tumour differentiation (*p* = .0161), lymph node metastasis (*p* = .006), advanced AJCC stage (*p* = .008) and more intensive treatment regimens (*p* = .006). Notably, high CD4^+^FOXP3^+^NOXA^+^ expression in the prospective cohort was also associated with lymph node metastasis (*p* = .0188), whereas NOXA expression alone showed no significant correlation with any clinicopathological variable in either cohort (Table S3).

**FIGURE 8:**
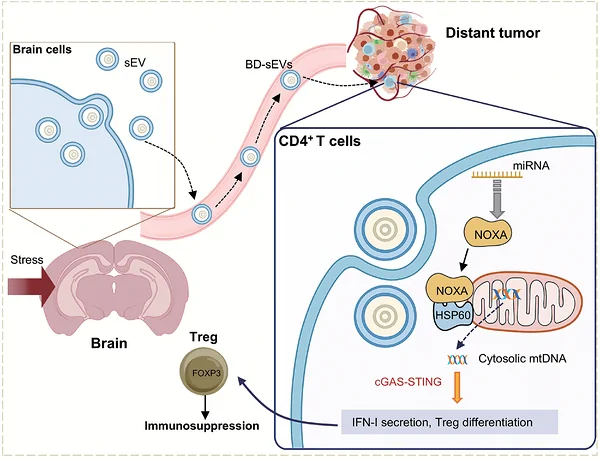
Schematic model of the chronic stress‐induced brain‐derived small extracellular vesicle (Stress‐BD‐sEV)‒phorbol‐12‐myristate‐13‐acetate‐induced protein 1 (NOXA)‒mitochondrial DNA (mtDNA)‒type I interferon (IFN‐I)‒regulatory T‐cell (Treg) differentiation axis. Chronic stress activates specific brain regions (hippocampal CA1–CA3 and caudate putamen [CPu]), promoting the secretion of brain‐derived small extracellular vesicles (BD‐sEVs) enriched with miR‐342‐3p, miR‐15a‐5p and miR‐381‐3p. These BD‐sEVs cross the blood–brain barrier and are internalised by CD4^+^ naïve T cells in the tumour microenvironment (TME). The microRNAs (miRNAs) upregulate NOXA, which interacts with heat shock protein 60 (HSP60) to induce mitochondrial dysfunction and mtDNA release. Cytosolic mtDNA activates the cyclic GMP‒AMP synthase (cGAS)‒stimulator of interferon genes (STING)‒IFN‐I pathway, driving Treg differentiation and immunosuppressive function, ultimately promoting colorectal cancer (CRC) progression.

In conclusion, we propose a mechanistic model in which chronic stress alters the secretion of L1CAM‐enriched sEVs (BD‐sEVs) consistent with a CNS origin, which deliver stress‐specific miRNAs to the CRC TME. Upon internalisation by CD4^+^ naïve T cells, these miRNAs upregulate NOXA, which interacts with HSP60 to induce mitochondrial dysfunction. The release of mtDNA activates the STING‒IFN‐I pathway, driving Treg differentiation and remodelling of the immunosuppressive TME and promoting CRC progression (Figure 8).

## 4 DISCUSSION

Chronic stress has been extensively documented to correlate significantly with adverse clinical outcomes in patients afflicted with diverse malignant tumours.^5^, ^7^ Although sustained activation of the HPA axis and the SNS constitute the principal pathway by which stress modulates tumour progression,^3^ the precise mechanisms through which the CNS orchestrates alterations in the peripheral tumour immune microenvironment across the blood–brain barrier remain inadequately elucidated. Our findings support a mechanistic framework delineated as follows: chronic stress remodels the cargo of BD‐sEVs, which carry miRNAs including miR‐342‐3p, miR‐15a‐5p and miR‐381‐3p. These miRNAs upregulate the proapoptotic protein NOXA, resulting in inhibition of HSP60, subsequent mtDNA release, activation of the cGAS‒STING–IFN‐I pathway, and ultimately promoting Treg differentiation. Depletion of Treg effectively reversed this pro‐tumourigenic effect, thereby confirming BD‐sEVs as pivotal mediators in this axis. These results unveil a cargo‐specific modality of neuroimmune communication that extends beyond classical stress hormone signalling, although direct comparative quantitation of BD‐sEV‐mediated versus hormone‐mediated Treg induction remains an important area for future investigation. Notably, chronic stress predominantly remodels the miRNA cargo composition of BD‐sEVs rather than increasing their particle number, with this qualitative alteration serving as the functional determinant for enhanced Treg differentiation (Figure S8B,C). The functional specificity of BD‐sEVs was further substantiated by concurrent analyses of PNS‐sEVs, which elicited only marginal STING activation and Treg differentiation (Figure S4E–I). Additionally, sEVs isolated from multiple distinct brain regions comparably induced STING activation and Treg differentiation (Figure S4C,D), indicating that c‐FOS activation signifies regional stress responsiveness rather than region‐specific sEV biogenesis. Furthermore, combined pharmacological blockade of HPA/SNS signalling (propranolol plus mifepristone) in an orthotopic CT26 model partially reversed the stress‐induced upregulation of Treg‐promoting BD‐sEV miRNA cargo and attenuated intratumoural NOXA–STING signalling in Tregs (Figures S2 and S3), providing in vivo evidence that the BD‐sEV–miRNA–NOXA–STING axis operates under intact upstream HPA/SNS signalling, with the partial reversal consistent with the involvement of parallel stress‐immune pathways.

NOXA, traditionally characterised as a proapoptotic BH3‐only protein, has garnered increasing recognition for its involvement in nonapoptotic functions such as metabolic reprogramming during T‐cell activation.^18^ Our study suggests a model in which stress‐triggered BD‐sEV‐derived miRNAs induce upregulation of NOXA, which in turn directly interacts with HSP60, suppressing its chaperone activity. This suppressive interaction compromises mitochondrial integrity and facilitates selective release of mtDNA. This mechanistic insight bridges two previously independent observations: cytoplasmic mtDNA‐driven activation of the cGAS–STING–IFN‐I axis,^22^, ^23^, ^45^ and mtDNA leakage consequent to HSP60 disruption,^46^ by identifying NOXA as the molecular nexus linking neural stress to mitochondrial perturbation in T cells. Importantly, this form of mitochondrial dysfunction is functionally distinct from conventional BAX/BAK‐mediated apoptosis. Subcellular fractionation experiments revealed selective mtDNA release without concomitant cytochrome c release or caspase‐3/7 activation (Figure S15). Moreover, overexpression of the anti‐apoptotic protein MCL‐1 rescued the pro‐apoptotic pathway but did not abrogate mtDNA release, STING activation, or Treg differentiation (Figure S15F–I), thereby genetically segregating NOXA's downstream effects into an MCL‐1‐dependent apoptotic branch and an MCL‐1‐independent, HSP60‐dependent branch responsible for Treg polarisation. Pharmacological exclusion of mPTP and VDAC pathways, together with partial contributions from BAX and ROS, supports the NOXA–HSP60 interaction as the functionally dominant mechanism (Figure S17). CD4^+^ T cells, specifically through Treg differentiation, represent the predominant functional cellular target, whereas CD8^+^ T cells and macrophages exhibited only partial and secondary responses (Figure S6F–K). This observation aligns with the lineage‐specific enrichment of *PMAIP1* (the gene encoding NOXA) in Treg across multiple malignancies (Figures 3E,F and S10). Gain‐ and loss‐of‐function experiments demonstrated that miR‐342‐3p, miR‐15a‐5p and miR‐381‐3p cooperatively elevate NOXA expression, with miR‐342‐3p making the most substantial contribution (Figures S8D–F and S9). Among the three stress‐responsive miRNAs identified, miR‐342‐3p emerged as the dominant contributor to NOXA upregulation and Treg differentiation, consistent with its established role in targeting Rictor/mTORC2 to regulate Treg lineage commitment. miR‐381‐3p and miR‐15a‐5p each induced partial but detectable effects in gain‐of‐function experiments, whereas no single miRNA mimic fully recapitulated the magnitude of Treg differentiation induced by intact Stress‐BD‐sEVs (Figure S8D–F). Complementary antagomir loss‐of‐function experiments confirmed the same functional hierarchy, with antagomir‐miR‐342‐3p producing the strongest attenuation of NOXA and Treg differentiation (Figure S9A–D). These findings support a multi‐miRNA cooperative model in which miR‐342‐3p provides the primary Treg‐polarising signal, with miR‐381‐3p and miR‐15a‐5p acting as complementary contributors that collectively drive the complete phenotype. Nonetheless, the precise molecular mechanism governing this regulation—whether via direct binding to the 3′UTR or through indirect pathways—remains to be elucidated.

The dual functionality of Treg in maintaining immune homeostasis and facilitating tumour immune evasion is well established.^47^ Notably, STING activation within CD4^+^ T cells has been shown to promote Treg differentiation via both canonical IFN‐I signalling and the MAPK‒CREB pathway,^24^ underscoring the multifaceted nature of STING‐mediated immune regulation. In the present study, we propose that chronic Stress‐BD‐sEVs augment both the abundance and immunosuppressive capacity of Treg through the NOXA–HSP60–mtDNA–cGAS‒STING signalling axis. Furthermore, enforced overexpression of HSP60 effectively antagonises this Treg‐promoting effect (Figure 4H), thereby implicating HSP60 as a promising therapeutic target for strategies aimed at modulating Treg function in the TME. Furthermore, pharmacological blockade of HPA/SNS signalling in a CT26 orthotopic model attenuated the stress‐induced upregulation of BD‐sEV miRNAs, Treg‐inducing capacity, and intratumoural STING pathway activation (Figures S2 and S3), providing in vivo evidence that the BD‐sEV–miRNA–NOXA–STING axis is regulated by upstream HPA/SNS signalling.

To address the ongoing controversy surrounding L1CAM as a specific marker for neuron‐derived EVs,^48^, ^49^, ^50^ we employed a comprehensive, multi‐dimensional orthogonal validation approach. Functional bioactivity assays revealed that plasma‐derived immunomodulatory effects were confined exclusively to the L1CAM‐positive fraction (Figure S13A–F), while sEVs isolated directly from brain tissue recapitulated the full functional signature of plasma‐derived BD‐sEVs with quantitatively comparable magnitudes (Figure S13G–K). Comparative global miRNA profiling indicated a high degree of correlation between BD‐sEVs and brain‐sEVs (i.e., sEVs isolated directly from brain tissue, whereas the BD‐sEVs used throughout this study were isolated from plasma and enriched via L1CAM) (Figure S12A). Brain‐enriched miRNAs (miR‐124‐3p, miR‐9‐5p and miR‐128‐1‐5p) were abundantly present in both BD‐sEVs and brain‐sEVs, yet were negligible or absent in PNS‐sEVs (Figure S12B). Proteomic analyses further confirmed the presence of neuronal markers such as synaptophysin and NeuN in both plasma BD‐sEVs and brain‐sEVs, with minimal contamination from GFAP indicative of astrocytic origin (Figure S11K). Collectively, these results, integrated with assessments by the International Society for Advancement of Alzheimer's Research and Treatment (ISTAART) as well as corroborative single‐EV analyses from orthogonal methodologies,^11^, ^13^, ^51^ provide convergent evidence supporting the neuronal origin of L1CAM‐enriched plasma sEVs. Nevertheless, it is important to acknowledge that L1CAM‐based enrichment currently remains an operational definition, pending definitive validation via in vivo lineage‐tracing techniques.

Epidemiological investigations have consistently demonstrated a significant association between chronic stress and cancer progression^2^, ^52^; however, the precise biological mechanisms underlying this relationship have remained insufficiently characterised. Our clinical data reveal that intratumoural expression levels of NOXA and FOXP3 correlate significantly with patients’ anxiety scores and serve as predictors of reduced overall survival. Specifically, patients exhibiting elevated preoperative anxiety corresponded with more advanced AJCC staging, whereas high co‐expression of CD4, FOXP3 and NOXA was linked to the presence of lymph node metastases (Table S3). Furthermore, patients with high HAMA scores exhibited decreased CD8^+^ T‐cell infiltration, diminished PD‐L1 expression, and a lower proportion of PD‐1–positive cells within the TME (Figure S18), indicative of an immunosuppressive milieu associated with heightened anxiety. These findings suggest that such patients may experience attenuated therapeutic efficacy from anti‐PD‐1/PD‐L1 immune checkpoint blockade. Consequently, combination therapies integrating psychological interventions with immunotherapy emerge as promising avenues warranting rigorous clinical evaluation. Collectively, our results provide a molecular framework substantiating clinical observations that psychoneurological factors critically influence cancer prognosis and advocate for the incorporation of psychological assessments into standard oncological management protocols.

This study acknowledges several limitations. First, the CRS model employed cannot fully replicate the multifaceted socio‐psychological stress experienced by cancer patients. A purely sequential experimental design entailing tumour establishment followed by stress induction is impractical because CRS protocols require a minimum of 21 days, whereas tumour‐bearing mice reach ethically mandated endpoints within approximately 17–23 days.^53^, ^54^, ^55^ Although serial OFT across two independent CRC models demonstrated that stress‐induced behavioural changes emerged only after tumour inoculation and reached significance at day 21 (Figures S1A–F and S2E–G), this temporal observation provides supportive evidence rather than a functional equivalent of a purely sequential paradigm. The BD‐sEV infusion experiments (Figure 1L–N) and Treg depletion studies (Figure 2N–Q) provide additional convergent support that stress acts on established tumours. Alternative paradigms such as unpredictable chronic mild stress (UCMS), although well validated,^56^ necessitate 4–6 weeks of exposure.^57^ and encompass stressors including food and water deprivation, which directly impact mitochondrial function and mtDNA release—the very pathway under investigation. Thus, CRS was selected to isolate neuroimmune signalling while minimising confounding metabolic effects. Additional validation in non‐metabolic stress models such as social defeat stress would enhance the generalisability of our findings. Second, although BD‐sEVs exhibited multi‐dimensional equivalence to brain tissue‐derived sEVs, definitive in vivo lineage tracing has yet to be performed; therefore, L1CAM‐based enrichment remains an operational surrogate marker consistent with, but not conclusive for, CNS origin. Third, attempts to inhibit specific brain regions via stereotaxic CPu microinjection were compromised by the introduction of independent surgical stress confounders. Open field behavioural assays confirmed that the microinjection procedure itself robustly altered anxiety‐like phenotypes (Figure S19), thereby precluding straightforward interpretation of direct CPu inhibition effects. Moreover, current methods for region‐specific sEV biogenesis inhibition (e.g., nSMase2 inhibitors, conditional knockout models, adeno‐associated viral vectors) lack the temporal and spatial precision compatible with the CRS experimental timeline. Fourth, the relatively limited sample size of our prospective cohort (*n* = 37) restricts statistical power for comprehensive multivariable adjustment, underscoring the necessity for larger, multicentre cohorts to validate circulating BD‐sEV miRNA signatures as clinically applicable biomarkers. Fifth, while the bidirectional genetic evidence from miRNA mimic gain‐of‐function and antagomir loss‐of‐function experiments supports a cooperative multi‐miRNA model, direct validation of miRNA–target interactions through dual‐luciferase reporter assays and mutagenesis of predicted binding sites has yet to be performed. The precise stoichiometry and relative contribution of each miRNA within the cooperative network therefore warrant further investigation.

Future studies should aim to: (1) establish the therapeutic potential of targeting the NOXA–HSP60 interaction using small‐molecule inhibitors or peptide‐based disruptors; (2) evaluate the efficacy of combined psychological intervention (e.g., stress reduction) with standard immunotherapy in preclinical CRC models; and (3) investigate whether circulating BD‐sEV miRNA profiles could serve as liquid biopsy biomarkers for identifying CRC patients who may benefit from psychoneuroimmunological interventions.

## 5 CONCLUSION

This study provides evidence for a brain‐to‐tumour immunosuppressive axis in which chronic stress alters the miRNA cargo of BD‐sEVs to drive NOXA‐mediated mitochondrial dysfunction, mtDNA release and cGAS‒STING‐dependent Treg differentiation. The identification of NOXA–HSP60 interaction as a potential therapeutic target, coupled with the clinical correlation between anxiety scores and intratumoural NOXA/FOXP3 expression, provides a mechanistic framework and potential biomarkers for psychoneuroimmunological cancer interventions.

## AUTHOR CONTRIBUTIONS

*Writing—review and editing, writing—original draft, visualisation, validation, methodology, investigation, data curation, conceptualisation and funding acquisition*: Huanhe Ni. *Methodology and investigation*: Yunyan Ling. *Investigation*: Ting Tang, Yuyang Liu, Zhen Chai, Shunjie Qing and Xujie Xiao. *Methodology*: Qiqiong Ding. *Data curation*: Dandan Zhao. *Writing—review and editing, methodology, conceptualisation and funding acquisition*: Hui Pan. *Writing—review and editing and resources*: Yan Lin. *Writing—review and editing, project administration, methodology, funding acquisition and conceptualisation*: Dehua Wu.

## CONFLICT OF INTEREST STATEMENT

The authors declare they have no conflicts of interest.

## ETHICS STATEMENT

Patients diagnosed with colorectal cancer were included in this study. All procedures were approved by the Medical Ethics Committee of Nanfang Hospital, Southern Medical University (NFEC‐2023‐465). All aspects of animal care and experimental procedures received approval from the Animal Care and Use Committee of Southern Medical University (SMUL2022189).

## Supporting information



Supporting Information

Supporting Information

## Data Availability

The proteome resources data reported in this paper have been deposited in the iProX (National Center for Protein Sciences, Beijing, China) (https://www.iprox.cn: accession no. IPX0015354001), ensuring full reproducibility. The raw sequence data reported in this paper have been deposited in the Genome Sequence Archive (Genomics, Proteomics & Bioinformatics 2025) in National Genomics Data Center (Nucleic Acids Res 2025), China National Center for Bioinformation/Beijing Institute of Genomics, Chinese Academy of Sciences (GSA: CRA037672 and CRA037646) that are publicly accessible at https://ngdc.cncb.ac.cn/gsa.
