# Supplementary material for: Chronic stress‐induced brain‐derived small extracellular vesicles promote colorectal cancer through Treg differentiation
Source: Clin Transl Med. 2026 Aug 21;16(8):e70756. doi: 10.1002/ctm2.70756 (PMC13494519; doi:10.1002/ctm2.70756)
Supplement: Supplementary file 1 [file CTM2-16-e70756-s001.docx]

**Supplementary Information**

**Chronic stress-induced brain-derived small extracellular vesicles promote colorectal cancer through Treg differentiation**

Huanhe Ni^1,2,#^, Yunyan Ling^1,#^, Ting Tang^3,#^, Yuyang Liu^1,#^, Zhen Chai^4^, Shunjie Qing^4^, Xujie Xiao^4^, Qiqiong Ding^5^, Dandan Zhao^5^, Hui Pan^5,*^, Yan Lin^2,*^,Dehua Wu^1,2,6,7,*^

1. Department of Radiation Oncology, Nanfang Hospital, Southern Medical University, Guangzhou, Guangdong Province, 510515, China

2. Department of Laboratory, Ganzhou People's Hospital, Gan Zhou, Jiang Xi, 341000, China

3. Department of Gynaecology, Ganzhou People's Hospital, Gan Zhou, Jiang Xi, 341000, China

4. Department of General Surgery, Nanfang Hospital, Southern Medical University, Guangzhou, Guangdong Province 510515, China.

5. Clinical Pharmacology Research Center, Peking Union Medical College Hospital, State Key Laboratory of Complex Severe and Rare Diseases, NMPA Key Laboratory for Clinical Research and Evaluation of Drug, Beijing Key Laboratory of Clinical PK & PD Investigation for Innovative Drugs, Chinese Academy of Medical Sciences & Peking Union Medical College, Beijing, 100730, China.

6. Guangdong Provincial Key Laboratory for Prevention and Control of Major Liver Diseases, Guangzhou, Guangdong Province 510515, China

7. Lead contact

\# These authors contributed equally

*Correspondence: panhui22@pumch.cn (H.P.), 19730809@sina.com (Y.L.), 18602062748@163.com (D.W.)

# 1. Supplementary Figures

## Figure S1. Validation of chronic stress-induced anxiety-like behaviours in the MC38 subcutaneous tumour model.

(A,B) Day 7 open field test (OFT) in the MC38 subcutaneous model. Central zone exploration time percentage, total distance, number of entries into the central zone, and rest times in control (Ctrl) and chronic restraint stress (CRS)-exposed C57BL/6 mice (*n* = 5 mice per group). No significant difference was detected at Day 7.

(C,D) Day 14 OFT. Stress group showed reduced central zone time percentage and entries into centre, and increased rest times, with total distance unchanged (*n* = 5 mice per group).

(E,F) Day 21 OFT. Stress group showed further reduction in central zone time percentage, reduced total distance, reduced entries into centre, and increased rest times compared with Ctrl (*n* = 5 mice per group).

(G) The sucrose preference test (SPT) was completed over 48 hours (24-hour adaptation period + 24-hour test period) to assess anhedonia. The data are presented as the percentage of sucrose intake relative to total fluid intake (n=5 per group).

Data are presented as mean ± SEM. **P* < 0.05, ***P* < 0.01, ****P* < 0.001; n.s., not significant. Unpaired two-tailed Student’s *t* test was used in (A–F).

## Figure S2. Validation of the stress-BD-sEVs-Treg axis in the CT26 orthotopic colorectal cancer model and pharmacological blockade of the HPA and SNS pathways.

(A) Schematic timeline of the CT26 orthotopic colorectal cancer model under CRS. CRS was initiated on Day 1; open field tests (OFT) were performed at Day 7 (followed by CT26 orthotopic cecal implantation after a rest interval), Day 14, and Day 21; daily propranolol (10 mg/kg, i.p.) and mifepristone (25 mg/kg, i.p.) were administered in the Stress+PROP+RU-486 group; CRS concluded at Day 1; endpoint at Day 21.

(B–D) CT26 orthotopic tumour-bearing mice were subjected to Ctrl, Stress, or Stress+PROP+RU-486 conditions. Representative images of orthotopic tumours (B), tumour weight at endpoint (C), and flow cytometry quantification of intratumoral FOXP3^+^ regulatory T (Treg) cell percentages (D) are shown (*n* = 5 mice per group).

(E–G) OFT quantification in the CT26 orthotopic model. Central zone exploration time percentage, total distance, entries in centre, and rest times at Day 7 (E), Day 14 (F), and Day 21 (G) in Ctrl and CRS-exposed (Stress) mice. Day 7 showed no significant differences; Day 14 stress mice showed reduced central zone time percentage and entries into centre, and increased rest times; Day 21 stress mice showed reduced central zone time percentage, total distance, and entries into centre, and increased rest times (*n* = 5 mice per group).

Data are presented as mean ± SEM. **P* < 0.05, ***P* < 0.01, ****P* < 0.001; n.s., not significant. One-way ANOVA was used in (B–D). Unpaired two-tailed Student’s *t* test was used in (E–G).

## Figure S3. Pharmacological dissection of the HPA/SNS-BD-sEV-miRNA-NOXA axis *in vivo*.

(A) Quantitative RT-PCR (qRT-PCR) analysis of miR-342-3p, miR-15a-5p, and miR-381-3p expression in L1CAM^+^ BD-sEVs isolated from plasma of Ctrl, Stress, and Stress+PROP+RU-486 (propranolol + mifepristone) mice, normalized to U6 small nuclear RNA (*n* = 3 mice per group). Stress significantly upregulated all three miRNAs; pharmacological blockade partially reversed this upregulation.

(B) Functional transfer assay. BD-sEVs isolated from the three groups were applied to CD4^+^ naïve T cells (10 μg/mL) for 72 h. Flow cytometry quantification of CD4^+^CD25^+^FOXP3^+^ Treg differentiation. Stress-BD-sEVs significantly promoted Treg differentiation; Stress+PROP+RU-486 BD-sEVs showed significantly attenuated Treg-inducing capacity (*n* = 3 independent experiments).

(C) Western blot analysis of NOXA protein levels in CD4^+^ naïve T cells treated with BD-sEVs from the three groups, with β-actin as loading control (*n* = 5 independent experiments).

(D,E) Enzyme-linked immunosorbent assay (ELISA) quantification of TGF-β (D) and IL-10 (E) concentrations in the culture supernatants of the functional transfer assay (*n* = 5 mice per group).

(F,G) Intratumoral Treg analysis. Tumour-infiltrating CD4^+^FOXP3^+^ Treg were gated for intracellular flow cytometry detection of NOXA protein levels (F) and phosphorylated TBK1 (p-TBK1, Ser172) and phosphorylated IRF3 (p-IRF3, Ser396) (G) (*n* = 5 mice per group). Stress group Treg showed the highest NOXA expression and STING pathway activation; Stress+PROP+RU-486 group showed partial attenuation.

Data are presented as mean ± SEM. **P* < 0.05, ***P* < 0.01, ****P* < 0.001; n.s., not significant. One-way ANOVA was used in (A–G).

## Figure S4. Characterisation of brain region-derived small extracellular vesicles (sEVs) and peripheral nerve-derived sEVs (PNS-sEVs).

(A) Immunostaining of c-FOS protein expression in brain sections from control and chronic restraint stress (CRS)-exposed mice in hippocampal regions (CA1, CA2, and CA3) and the caudate putamen (CPu).

(B) sEVs were isolated from the hippocampal CA1–CA3 region, the CPu region, and other brain regions of stressed mice. Western blot analysis of sEV markers (TSG101, CD63, and L1CAM) and loading control (β-actin). All three brain region-derived sEVs expressed TSG101, CD63, and L1CAM.

(C,D) CD4^+^ naïve T cells were treated with sEVs (10 μg/mL) from the indicated brain regions for 72 h. (C) Western blot analysis of phospho-TBK1 (p-TBK1), total TBK1, phospho-IRF3 (p-IRF3), total IRF3, and FOXP3, with β-actin as loading control. sEVs from all three brain regions activated STING signalling. (D) Flow cytometry quantification of CD4^+^CD25^+^FOXP3^+^ regulatory T (Treg) cell differentiation. All three brain region-derived sEVs promoted Treg differentiation to a comparable extent (*n* = 3 independent experiments).

(E) Schematic diagram of the workflow for isolating brain-derived small extracellular vesicles (BD-sEVs) from peripheral blood via anti-L1CAM antibody-based immunoprecipitation.

(F) Protein mass of PNS-sEVs enriched by anti-p75-NTR antibody from plasma of Ctrl and CRS-exposed mice, quantified by BCA assay. No significant difference was observed between groups (*n* = 5 mice per group).

(G) Western blot analysis of sEV markers (CD63, TSG101, L1CAM, and p75-NTR) in Stress-BD-sEVs and Stress-PNS-sEVs. Stress-BD-sEVs showed high CD63/TSG101/L1CAM and low p75-NTR; Stress-PNS-sEVs showed high CD63/TSG101/p75-NTR and low L1CAM, confirming distinct molecular fingerprints.

(H,I) CD4^+^ naïve T cells were treated with Ctrl-BD-sEVs, Stress-BD-sEVs, Ctrl-PNS-sEVs, or Stress-PNS-sEVs (10 μg/mL) for 72 h. (H) Western blot analysis of phospho-TBK1 (p-TBK1), total TBK1, phospho-IRF3 (p-IRF3), and total IRF3. Stress-BD-sEVs potently activated STING signalling, whereas Stress-PNS-sEVs induced only marginal effects. (I) Flow cytometry quantification of CD4^+^CD25^+^FOXP3^+^ regulatory T (Treg) cell differentiation (*n* = 3 independent experiments).

Data are presented as mean ± SEM. **P* < 0.05, ***P* < 0.01, ****P* < 0.001; n.s., not significant. Unpaired two-tailed Student’s *t* test was used in (F) and (I)

## Figure S5. Functional validation of BD-sEV effects on viability and the NOXA-HSP60-cGAS-STING pathway in colorectal cancer (CRC) cells.

(A-C) *In vitro* functional experiments. MC38 (A) and CT26 (B) mouse CRC cells were treated with Ctrl-BD-sEVs or Stress-BD-sEVs for 72 h, and cell viability was determined using the CCK-8 assay. No significant difference was detected (*n* = 5 independent experiments). (C) The apoptosis rate of MC38 cells was quantified via Annexin V-PI staining and flow cytometry. No significant difference was detected (*n* = 5 independent experiments).

(D) Western blot analysis of NOXA, HSP60, phosphorylated TBK1 (p-TBK1), and phosphorylated IRF3 (p-IRF3) in MC38 and CT26 CRC cell lines treated with Ctrl-BD-sEVs or Stress-BD-sEVs.

(E) Quantitative PCR (qPCR) quantification of cytosolic mitochondrial DNA (mtDNA) levels (ND1, ND5) in MC38 and CT26 cells (*n* = 3 independent experiments).

(F) Enzyme-linked immunosorbent assay (ELISA) quantification of IFN-β secretion in cell culture supernatants (*n* = 3 independent experiments).

Data are presented as mean ± SEM. **P* < 0.05, ***P* < 0.01, ****P* < 0.001; n.s., not significant. Unpaired two-tailed Student’s *t* test was used in (A-F).

## Figure S6. Gating strategies, dose-response, BD-sEVs uptake inhibition, and immune cell subset analysis.

(A) Gating strategy for flow cytometry detection of CD4^+^CD25^+^FOXP3^+^ regulatory T (Treg) cells in the BD-sEV-induced Treg differentiation *in vitro* experiment.

(B) CD4^+^ naïve T cells were treated with increasing doses of Stress-BD-sEVs (1, 10, and 100 μg/mL) for 72 h. Treg differentiation was quantified by flow cytometry, showing a dose-dependent increase that plateaued at 10 μg/mL (*n* = 3 independent experiments).

(C) CD4^+^ naïve T cells were treated with Stress-BD-sEVs in the presence of the sEV uptake inhibitor heparin or vehicle control for 72 h. Flow cytometry quantification of CD4^+^CD25^+^FOXP3^+^ Treg differentiation. Heparin significantly reduced Treg differentiation (*n* = 3 independent experiments).

(D,E) CD45^+^CD8^+^ IFN-γ^+^ T cell frequencies in tumour-infiltrating lymphocytes from the CRS model (D) and the BD-sEV infusion model (E), detected by flow cytometry. CD45^+^CD8^+^ IFN-γ^+^ T cell frequencies were reduced in both models (*n* = 5 mice per group).

(F,G) CD4^+^ T cells were treated with Ctrl-BD-sEVs or Stress-BD-sEVs for 72 h. Representative flow cytometry plots (F) and quantification of CD4^+^CD25^+^FOXP3^+^ Treg differentiation (G) are shown. Stress-BD-sEVs significantly promoted Treg differentiation, with no significant effect on IL-4 or IL-17 production (*n* = 3 independent experiments).

(H,I) CD8^+^ T cells were treated with Ctrl-BD-sEVs or Stress-BD-sEVs for 72 h. Representative flow cytometry plots of effector markers (IFN-γ, Granzyme B) and exhaustion markers (PD-1, TIM-3) (H) and quantification of positive percentages (I) are shown. Stress-BD-sEV treatment increased IFN-γ, Granzyme B, and TIM-3 expression; PD-1 showed no significant difference (*n* = 3 independent experiments).

(J,K) Bone marrow-derived macrophages were treated with Ctrl-BD-sEVs or Stress-BD-sEVs for 72 h. Representative flow cytometry plots of M1 (CD80^+^MHC-II) and M2 (CD80^-^CD206) activation markers (J) and quantification of positive percentages (K) are shown. Stress-BD-sEVs increased M1 markers, with no significant change in M2 markers (*n* = 3 independent experiments).

Data are presented as mean ± SEM. **P* < 0.05, ***P* < 0.01, ****P* < 0.001; n.s., not significant. One-way ANOVA was used in (B). Unpaired two-tailed Student’s *t* test was used in (C), (D–K).

## Figure S7. Treg depletion in the direct chronic restraint stress (CRS) model.

(A) Schematic of the CRS and CD25 Ab depletion experimental protocol.

(B) Representative images of tumours from Stress+IgG and Stress+CD25 Ab groups.

(C,D) Tumour volume growth curves (C) and final tumour weight at endpoint (D) in CRS-exposed MC38 tumour-bearing mice treated with CD25 Ab depletion antibody or isotype control IgG. CD25 Ab treatment significantly attenuated tumour growth and reduced tumour weight (*n* = 5 mice per group).

(E,F) Tumour-infiltrating immune cells were analysed by multicolour flow cytometry with t-SNE dimensionality reduction (E), and the proportions of immune cell subsets were quantified (F). Treg were significantly reduced; CD8^+^ T cell proportions were relatively restored; other subsets showed no significant changes (*n* = 5 mice per group).

(G) CD8^+^ T cell effector and exhaustion marker analysis. IFN-γ, Granzyme B, PD-1, and TIM-3 expression in tumour-infiltrating CD8^+^ T cells. CD25 Ab treatment increased IFN-γ and Granzyme B expression and decreased PD-1 and TIM-3 levels (*n* = 5 mice per group).

Data are presented as mean ± SEM. **P* < 0.05, ***P* < 0.01, ****P* < 0.001. Unpaired two-tailed Student’s *t* test was used in (C,D), (F–G).

## Figure S8. Dissection of BD-sEV functional cargo and miRNA gain-of-function and loss-of-function validation.

(A) Quantitative RT-PCR (qRT-PCR) validation of miR-342-3p, miR-15a-5p, and miR-381-3p expression in Ctrl-BD-sEVs and Stress-BD-sEVs, normalized to U6 small nuclear RNA. All three miRNAs were significantly upregulated in Stress-BD-sEVs (*n* = 3 independent experiments).

(B,C) Stress-BD-sEVs were pre-treated with protease K (50 μg/mL, 30 min, 37°C, inactivated with 5 mM PMSF) or RNase A (10 μg/mL, 30 min, 37°C, with RNase inhibitor) and applied to CD4^+^ naïve T cells (10 μg/mL) for 72 h alongside untreated Ctrl-BD-sEVs and Stress-BD-sEVs. (B) Flow cytometry quantification of CD4^+^CD25^+^FOXP3^+^ regulatory T (Treg) cell differentiation. Protease K treatment did not abolish the enhanced Treg-inducing capacity of Stress-BD-sEVs; RNase A treatment largely abrogated it (*n* = 3 independent experiments). (C) Western blot analysis of NOXA protein levels, with β-actin as loading control.

(D) CD4^+^ naïve T cells were electroporated with microRNA (miRNA) mimics (miR-342-3p, miR-381-3p, miR-15a-5p) or mimic negative control (Mimic NC) and cocultured with Ctrl-BD-sEVs for 72 h, with Stress-BD-sEVs + mimic-NC as positive control. Western blot analysis of NOXA protein levels, with β-actin as loading control. miR-342-3p produced the strongest NOXA upregulation.

(E) Flow cytometry quantification of CD4^+^CD25^+^FOXP3^+^ Treg differentiation after miRNA mimic electroporation. miR-342-3p showed the strongest induction; miR-381-3p and miR-15a-5p each induced partial effects. No single miRNA fully recapitulated Stress-BD-sEV-induced Treg differentiation (*n* = 3 independent experiments).

(F) qRT-PCR validation of miRNA mimic electroporation efficiency. Three independent bar graphs confirming successful overexpression of miR-15a-5p, miR-342-3p, and miR-381-3p after respective mimic electroporation, normalized to U6 (*n* = 3 independent experiments).

Data are presented as mean ± SEM. **P* < 0.05, ***P* < 0.01, ****P* < 0.001; n.s., not significant. Unpaired two-tailed Student’s *t* test was used in (A). One-way ANOVA was used in (B), (E–K).

## Figure S9. Loss-of-function validation of stress-responsive miRNAs by antagomir knockdown in BD-sEV-mediated Treg differentiation.

(A) Schematic of the antagomir loss-of-function experiment. CD4^+^ naïve T cells were electroporated with antagomir negative control (antagomir-NC), antagomir-miR-342-3p, antagomir-miR-381-3p, or antagomir-miR-15a-5p, followed by coculture with Ctrl-BD-sEVs or Stress-BD-sEVs for 72 h. Western blot analysis of NOXA protein levels after antagomir treatment, with β-actin as loading control. Antagomir-miR-342-3p produced the strongest attenuation of NOXA protein levels; antagomir-miR-381-3p and antagomir-miR-15a-5p produced partial attenuation.

(B) qRT-PCR assessment of antagomir specificity. Each antagomir selectively reduced its target miRNA level without affecting non-target miRNAs, normalized to U6. Note: antagomirs silence miRNA by competitive binding rather than degradation; the qPCR signal reflects partial thermal dissociation of the antagomir-miRNA duplex during reverse transcription and does not reflect functional inhibition (*n* = 3 independent experiments).

(C) Flow cytometry quantification of CD4^+^CD25^+^FOXP3^+^ Treg differentiation after antagomir treatment. Antagomir-miR-342-3p produced the strongest attenuation; antagomir-miR-381-3p and antagomir-miR-15a-5p produced partial attenuation. No single antagomir completely abolished the Stress-BD-sEV phenotype (*n* = 3 independent experiments).

(D) Annexin V/propidium iodide (PI) apoptosis analysis showing no significant difference across antagomir treatment groups, confirming that antagomir-mediated miRNA silencing does not affect cell viability (*n* = 3 independent experiments).

Data are presented as mean ± SEM. **P* < 0.05, ***P* < 0.01, ****P* < 0.001; n.s., not significant. One-way ANOVA was used in (A-D).

## Figure S10. *PMAIP1* expression in regulatory T (Treg) cells across multiple tumour types.

(A) Table showing the intersection of genes highly expressed in tumour-infiltrating Treg across 6 independent single-cell RNA sequencing (scRNA-seq) datasets of gastrointestinal tumours.

(B) Validation based on the ImmuNexUT database showing that *PMAIP1* is highly expressed specifically in the Fr.II effector Treg (eTreg) subset (phenotype: CD3+CD4^+^CD8-CD25^+^+CD45RA^-^), which has potent immunosuppressive function.

(C) Violin plot showing *PMAIP1* expression in each subset of tumour-infiltrating lymphocytes (TILs) based on a published colorectal cancer (CRC) scRNA-seq dataset.

(D–L) Pan-cancer analysis based on the Tumor Immune Single-cell Hub (TISCH) database showing *PMAIP1* expression in Treg subsets across multiple tumour types, including non-small cell lung cancer (NSCLC) (D), liver hepatocellular carcinoma (LIHC) (E), colorectal cancer (CRC) (F), breast invasive carcinoma (BRCA) (G), glioblastoma multiforme (glioma) (H), and uterine corpus endometrial carcinoma (UCEC) (I–L).

## Figure S11. Characterisation of brain-tissue-derived small extracellular vesicles (brain-sEVs) and functional enrichment analysis of microRNA (miRNA) target genes.

(A–C) Characterisation of sEVs isolated from whole mouse brain tissue (brain-sEVs). (A) Transmission electron microscopy (TEM) image showing the typical cup-shaped morphology (scale bar = 100 nm). (B) Flow cytometry detection of sEV markers. (C) Nanoparticle tracking analysis (NTA) for quantification of brain-sEV concentration.

(D) Western blot analysis of HSP60 total protein levels in CD4^+^ naïve T cells treated with Ctrl-BD-sEVs or Stress-BD-sEVs (10 μg/mL) for 72 h, with β-actin as loading control. No significant difference was observed between groups (*n* = 3 independent experiments).

(E–G) Gene Ontology (GO) enrichment analysis of target genes of differentially expressed miRNAs in brain tissue-derived sEVs: (E) biological processes (BPs), (F) cellular components (CCs), and (G) molecular functions (MFs).

(H–J) Venn diagrams showing the overlapping pathways in the GO functional categories between the miRNA target genes of BD-sEVs and those of whole-brain-derived sEVs: (H) biological processes (BPs), (I) cellular components (CCs), and (J) molecular functions (MFs).

(K) Western blot comparison of L1CAM-enriched plasma sEVs (BD-sEVs) and brain-tissue-derived sEVs (brain-sEVs) for L1CAM, neuronal markers (synaptophysin, NeuN), glial marker GFAP, and canonical EV markers (CD63, TSG101). Equal protein input. Both sEV preparations shared neuronal markers and canonical EV markers, with low GFAP in both.

Data are presented as mean ± SEM. n.s., not significant. Unpaired two-tailed Student’s *t* test was used in (D).

## Figure S12. Multi-dimensional validation of the neuronal origin of L1CAM-enriched plasma sEVs.

(A) Spearman correlation heatmap of global miRNA expression profiles across Ctrl-BD-sEVs, Stress-BD-sEVs, Ctrl-brain-sEVs, and Stress-brain-sEVs (n = 3 per group).

(B) Brain-enriched miRNA fingerprint (miR-124-3p, miR-9-5p, miR-128-3p; left of dashed line) in BD-sEVs, brain-sEVs, quantified by miRNA-seq .

(C) ceRNA network centered on stress-responsive miRNAs. Competitive endogenous RNA (ceRNA) network constructed from miRNA, mRNA, and lncRNA sequencing data. Red squares = core miRNAs (miR-342-3p, miR-15a-5p, miR-381-3p); blue circles = lncRNAs with anti-correlated expression (Pearson); green rounded squares = mRNA targets with anti-correlated expression to their regulatory miRNA. Solid red edges = miRNA-mediated inhibition; dashed blue edges = positive lncRNA–mRNA correlation (Pearson), indicative of ceRNA competition. The network comprises 41 nodes (3 miRNAs, 15 lncRNAs, 23 mRNAs) and 124 edges. Key miRNA–target pairs include miR-381-3p→Mdm2, miR-342-3p→Ifit1, and miR-15a-5p→Mcl1, linking stress-responsive miRNAs to p53 signaling, interferon response, and apoptosis regulation.

(D) KEGG pathway enrichment analysis of the 23 mRNAs in the ceRNA network. Bar length represents the number of enriched genes per pathway. Top enriched pathways include PI3K-Akt signaling (11 genes), Pathways in cancer (11 genes), and HIF-1 signaling (9 genes), consistent with the known roles of miR-342-3p, miR-15a-5p, and miR-381-3p in stress response, immune regulation, and cell survival. Data are expressed as mean ± SEM. **P* < 0.05, ***P* < 0.01, ****P* < 0.001. One-way ANOVA was used in (B) and (D).

## Figure S13. Functional depletion and brain-tissue equivalence validation of L1CAM-enriched BD-sEVs.

(A) Schematic of the anti-L1CAM immunoprecipitation experiment. Plasma sEVs were incubated with biotinylated anti-L1CAM antibody and streptavidin magnetic beads. The immunoprecipitation pellet (L1CAM^+^ BD-sEVs) and flow-through (L1CAM-depleted sEVs) were collected for parallel functional assays.

(B) Flow cytometry quantification of CD4^+^CD25^+^FOXP3^+^ Treg differentiation after treatment with Ctrl-BD-sEVs, Stress-BD-sEVs, or Stress L1CAM-depleted sEVs (10 μg/mL) for 72 h. L1CAM-depleted sEVs lost Treg-inducing capacity (*n* = 3 independent experiments).

(C) Western blot validation of sEV markers (L1CAM, synaptophysin, NeuN, CD63, TSG101, β-actin) in the three sEV preparations. L1CAM, synaptophysin, and NeuN were detected only in Ctrl-BD-sEVs and Stress-BD-sEVs; CD63, TSG101, and β-actin were detected in all three groups.

(D) Western blot analysis of NOXA, phosphorylated TBK1 (p-TBK1), total TBK1, phosphorylated IRF3 (p-IRF3), total IRF3, and β-actin in CD4^+^ naïve T cells treated with the three sEV preparations.

(E) Quantitative PCR (qPCR) quantification of cytosolic mitochondrial DNA (mtDNA) levels (ND1, ND5) (*n* = 3 independent experiments).

(F) Enzyme-linked immunosorbent assay (ELISA) quantification of IFN-β secretion (*n* = 3 independent experiments).

(G) Schematic of the brain tissue sEV extraction and parallel functional comparison experiment.

(H) Flow cytometry quantification of CD4^+^CD25^+^FOXP3^+^ Treg differentiation after treatment with Stress brain-sEVs or Stress-BD-sEVs for 72 h. Stress brain-sEVs promoted Treg differentiation at a magnitude comparable to Stress-BD-sEVs (*n* = 3 independent experiments).

(I) Western blot analysis of NOXA, p-TBK1, total TBK1, p-IRF3, total IRF3, and β-actin in CD4^+^ naïve T cells treated with Ctrl-BD-sEVs, Stress-BD-sEVs, or Stress brain-sEVs.

(J) qPCR quantification of cytosolic mtDNA (ND1, ND5) (*n* = 3 independent experiments).

(K) ELISA quantification of IFN-β secretion (*n* = 3 independent experiments).

Data are presented as mean ± SEM. **P* < 0.05, ***P* < 0.01, ****P* < 0.001; n.s., not significant. One-way ANOVA was used in (B), (D), (E), (F), (H), (I), (J), and (K).

## Figure S14. Mass spectrometry analysis of NOXA-binding proteins and comparison with brain-tissue-derived sEVs.

(A) Gene Ontology (GO) enrichment analysis of proteins obtained by co-immunoprecipitation (Co-IP) with NOXA from CD4^+^ naïve T cells treated with Ctrl-BD-sEVs or Stress-BD-sEVs.

(B) Bar graph showing the differences in the expression of proteins related to various subcellular structures associated with NOXA-interacting components between the Stress-BD-sEV group and the Ctrl-BD-sEV group.

(C) List of the top 10 NOXA-binding proteins ranked by abundance determined by mass spectrometry analysis.

(D) Flow cytometry verified that HSP60 was successfully knocked down by shRNA-mediated lentiviral knockdown in CD4^+^ T cells.

(E) Flow cytometry verified that NOXA and HSP60 was successfully over expression by lentivirus in CD4^+^ T cells.

## Figure S15. Mechanistic dissection of non-apoptotic mitochondrial stress and MCL-1-independent Treg polarization downstream of NOXA.

(A) Western blot validation of subcellular fractionation purity: cytosolic (Cyto) fractions probed for cytochrome c, COX IV, and β-actin.

(B) qPCR quantification of cytosolic mtDNA (ND1, ND5) in CD4^+^ naïve T cells treated with Ctrl-BD-sEVs or Stress-BD-sEVs (n = 3 biological replicates per group).

(C-E) CD4^+^ naïve T cells were transduced with sh-NC or sh-NOXA and treated with Stress-BD-sEVs (10 ug/mL, 72 h), with Ctrl-BD-sEV treatment as baseline control. (C) Annexin V/PI apoptosis analysis (n = 3 biological replicates per group).

(D) Flow cytometry quantification of CD4^+^CD25^+^FOXP3^+^ regulatory T (Treg) cell differentiation (n = 3 biological replicates per group).

(E) CellEvent Caspase-3/7 Green activation assay (n = 3 biological replicates per group). (F-I) CD4^+^ naïve T cells overexpressing NOXA (OE-NOXA) were further transduced with MCL-1 overexpression (OE-MCL-1) or empty vector control (OE-NC). (F) Cytosolic mtDNA (ND1, ND5) quantified by qPCR (n = 3 biological replicates per group).

(G) Annexin V/PI apoptosis analysis (n = 3 biological replicates per group).

(H) Western blot analysis of STING pathway activation (p-TBK1, TBK1, p-IRF3, IRF3), with β-actin as loading control.

(I) Flow cytometry quantification of CD4^+^CD25^+^FOXP3^+^ Treg cell differentiation (n = 3 biological replicates per group).

(J) Flow cytometry verified that NOXA and MCL1 was successfully over expression by lentivirus in CD4^+^ T cells.

Data are expressed as mean plus/minus SEM. **P <* 0.05, ***P <* 0.01, ****P <* 0.001; n.s., not significant. Unpaired two-tailed Student t test was used in (B). One-way ANOVA was used in (C-I).

## Figure S16. Biochemical mechanism of NOXA-mediated negative regulation of HSP60 chaperone activity.

(A) Cycloheximide (CHX) chase assay. CD4^+^ naïve T cells infected with OE-NC or OE-NOXA lentivirus were treated with CHX (50 μg/mL) for 0, 24, and 48 h. HSP60, NOXA, and β-actin protein levels were detected by Western blotting. HSP60 protein levels remained stable over 48 h in both groups, with no detectable difference in decay rates (*n* = 3 independent experiments).

(B) HSP60 ubiquitination assay in 293T cells. Cells were cotransfected with OE-NC or OE-NOXA, treated with or without MG132 (10 μm, 6 h), and subjected to immunoprecipitation (IP) with anti-HSP60 or IgG control antibody. HSP60 ubiquitination levels were comparable between OE-NC and OE-NOXA groups.

(C) Citrate synthase (CS) thermal aggregation assay. IP-purified HSP60 from OE-NC or OE-NOXA CD4^+^ naïve T cells was incubated with CS (0.15 μm) at 43°C for 45 min. Light scattering at 500 nm was recorded every 60 s. IP-HSP60(OE-NOXA) showed significantly reduced suppression of CS aggregation compared with IP-HSP60(OE-NC) (*n* = 3 independent experiments).

Data are presented as mean ± SEM. **P* < 0.05, ***P* < 0.01, ****P* < 0.001. One-way ANOVA was used in (C).

## Figure S17. Pharmacological epistasis of mitochondrial DNA (mtDNA) release pathways.

(A) Schematic of the pharmacological inhibitor experiment. CD4^+^ naïve T cells were treated with Ctrl-BD-sEVs+DMSO, Stress-BD-sEVs+DMSO, Stress-BD-sEVs+NIM811 (5 μm, mPTP inhibitor), Stress-BD-sEVs+BAI1 (5 μm, BAX inhibitor), Stress-BD-sEVs+VBIT-4 (10 μm, VDAC inhibitor), or Stress-BD-sEVs+NAC (5 mM, ROS scavenger) for 72 h.

(B) Quantitative PCR (qPCR) quantification of cytosolic mtDNA (ND1, ND5). NIM811 and VBIT-4 produced no significant attenuation; BAI1 and NAC produced partial attenuation (*n* = 3 independent experiments).

(C) Western blot analysis of phosphorylated TBK1 (p-TBK1), total TBK1, phosphorylated IRF3 (p-IRF3), and total IRF3. p-TBK1.

(D) Enzyme-linked immunosorbent assay (ELISA) quantification of IFN-β secretion. NIM811 and VBIT-4 produced no significant attenuation; BAI1 and NAC produced partial attenuation (*n* = 3 independent experiments).

(E) Flow cytometry quantification of CD4^+^CD25^+^FOXP3^+^ regulatory T (Treg) cell differentiation. NIM811 and VBIT-4 produced no significant attenuation; BAI1 and NAC produced partial attenuation (*n* = 3 independent experiments).

Data are presented as mean ± SEM. **P* < 0.05, ***P* < 0.01, ****P* < 0.001; n.s., not significant. One-way ANOVA was used in (B), (D), and (E).

## Figure S18. Multicolour immunofluorescence staining of immune checkpoint molecules in human colorectal cancer tissues.

(A) Human colorectal cancer (CRC) patient specimens stratified by Hamilton Anxiety Rating Scale (HAMA) scores (low vs. high). Multicolour immunofluorescence (IF) staining for DAPI, CD8, PD-L1, and PD-1. Representative images are shown.

(B) Quantification of PD-L1, PD-1, and CD8 expression in low-HAMA vs. high-HAMA patient tissues (n = 10 biological replicates per group).

Data are expressed as mean plus/minus SEM. **P* < 0.05, ****P* < 0.001. Unpaired two-tailed Student t test was used in (B). Scale bar = 50 um.

## Figure S19. Open field test assessment after CPu stereotaxic microinjection surgery in control and stressed mice

(A) Stereotaxic atlas showing CPu injection coordinates.

(B) Experimental scheme: mice received CPu saline (vehicle) microinjection or muscimol injection on Day 1; open field tests (OFT) were performed on Day 7, and Day 14. Six groups were compared: non-surgical Ctrl (Normal Ctrl), non-surgical Stress (Normal Stress), Stereotactic injection vehicle Ctrl, Stereotactic injection vehicle Stress, Stereotactic injection muscimol Ctrl, and Stereotactic injection muscimol Stress. (C,D) Representative OFT tracking plots and quantification on Day 7, including central zone time (%), total distance (cm), entries in center, and rest time (n = 3 biological replicates per group).

(E,F) Representative OFT tracking plots and quantification on Day 14 for the same metrics (n = 3 biological replicates per group). Data are expressed as mean ± SEM. **P <* 0.05, ***P <* 0.01, ****P <* 0.001. Two-way ANOVA was used in (C)-(F).

# 2. Supplementary Method

**2.1 Orthotopic CRC model**

BALB/c mice were subjected to the CRS protocol (Day 1 to Day 21) as described in Section 2.3. CT26 colon cancer cells were surgically implanted into the cecal wall on Day 7 under isoflurane anaesthesia. Mice were randomly assigned to three groups: control (Ctrl), chronic restraint stress (Stress), and Stress plus pharmacological blockade of the HPA and SNS pathways (Stress+PROP+RU-486). In the Stress+PROP+RU-486 group, propranolol (10 mg/kg, i.p.) and mifepristone (25 mg/kg, i.p.) were administered daily beginning on Day 1. Open field tests were performed at Day 0 (baseline), Day 7, Day 14, and Day 21. The experimental endpoint was Day 27. Tumour weight and volume were measured at endpoint, and intratumoral immune cell proportions were analysed by flow cytometry.

## 2.2 Enzyme-linked immunosorbent assay (ELISA)

The cell supernatants were collected after the medium was replaced with fresh medium and filtered through 0.45 μm filters. The IL-10 and TGF-β concentrations were subsequently measured using ELISA kits (Dakewe, China) per the manufacturer’s guidelines.

## 2.3 Western blotting

Cells were washed three times with PBS and lysed on ice with RIPA buffer (Beyotime) for 30 min. After centrifugation, the supernatants were collected, and the protein concentration was determined via a BCA assay (Beyotime). Ten micrograms of protein was separated by 10% SDS–PAGE, transferred to PVDF membranes (Millipore), blocked with 5% milk in TBS, and incubated with primary antibodies overnight at 4°C. After being washed with TBS-T, the membranes were incubated with peroxidase-conjugated secondary antibodies (1:1000; Beyotime) for 1 h at 20°C, after which the bands were visualized via ECL (FDbio-pico). The antibodies used are listed in Supplementary Table 4.

## 2.4 Quantitative reverse transcription–polymerase chain reaction (RT–PCR)

Total mRNA was extracted with TRIzol reagent (Invitrogen) and reverse-transcribed into cDNA using a PrimeScript RT–PCR Kit (TaKaRa). Quantitative RT–PCR was performed with SYBR qPCR Master Mix (Tsingke) per the manufacturer’s instructions.

## 2.5 Cargo dissection of sEVs

To dissect the functional contribution of cargo molecules, Stress-BD-sEVs were pre-treated with protease K (50 μg/mL, 30 min, 37°C, inactivated with 5 mM PMSF) or RNase A (10 μg/mL, 30 min, 37°C, with RNase inhibitor). After treatment, sEVs were applied to CD4^+^ naïve T cells at equal input doses (10 μg/mL final concentration) alongside untreated Ctrl-BD-sEVs and Stress-BD-sEVs.

## 2.6 Quantitative real-time PCR of exosomal miRNAs

Total RNA was extracted from sEV pellets using TRIzol reagent (Invitrogen). Reverse transcription was performed using a Bulge-Loop miRNA qRT-PCR Starter Kit (RiboBio) per the manufacturer’s instructions. Quantitative PCR was performed with SYBR qPCR Master Mix (Tsingke) on a QuantStudio 3 system (Applied Biosystems). The expression levels of miR-342-3p, miR-15a-5p, and miR-381-3p were normalized to U6 small nuclear RNA. This protocol was adapted from previous study ^[34]^.

## 2.7 miRNA mimic gain-of-function and antagomir loss-of-function assays

For miRNA mimic gain-of-function experiments, CD4^+^ naïve T cells were electroporated with synthetic miRNA mimics (miR-342-3p, miR-381-3p, miR-15a-5p, or mimic NC; RiboBio) using a Nucleofector system (Lonza) per the manufacturer’s protocol. Cells were co-treated with Ctrl-BD-sEVs or Stress-BD-sEVs (10 μg/mL) for 72 h. NOXA protein levels were assessed by Western blotting, and Treg differentiation was analysed by flow cytometry. A Stress-BD-sEV + mimic-NC group served as the positive control.

For antagomir loss-of-function experiments, CD4^+^ naïve T cells were electroporated with antagomir-NC, antagomir-miR-342-3p, antagomir-miR-381-3p, or antagomir-miR-15a-5p (RiboBio), activated with anti-CD3/CD28 plus IL-2, and co-treated with Ctrl-BD-sEVs or Stress-BD-sEVs (10 μg/mL) for 72 h. NOXA protein levels, Treg differentiation, and cell viability (Zombie NIR) were analysed.

## 2.8 Myeloid cell leukemia 1 (MCL-1) overexpression rescue assay

Full-length MCL-1 was subcloned into the pSin vector. CD4^+^ naïve T cells were co-transduced with OE-NOXA and OE-MCL-1 or empty vector control lentiviruses as described in Section 2.14. Cells were analysed for cytosolic mtDNA (qPCR), apoptosis (Annexin V/PI), STING pathway activation (p-TBK1, p-IRF3 Western blot), and Treg differentiation (flow cytometry).

## 2.9 NOXA-HSP60 biochemical mechanism assays

For cycloheximide (CHX) chase assays, CD4^+^ naïve T cells transduced with OE-NC or OE-NOXA lentivirus for 48 h were treated with cycloheximide (50 μg/mL) and harvested at 0, 24, and 48 h. HSP60 protein levels were assessed by Western blotting.

For HSP60 ubiquitination assays, 293T cells co-transfected with empty vector (OE-NC) or Flag-NOXA were treated with MG132 (10 μm, 6 h). HSP60 was immunoprecipitated using anti-HSP60 antibody, and ubiquitination was detected with anti-poly-ubiquitin antibody (FK2). IP efficiency was confirmed by re-blotting with anti-HSP60.

For citrate synthase (CS) thermal aggregation assays, HSP60 was immunoprecipitated from OE-NC or OE-NOXA CD4^+^ naïve T cells. CS (0.15 μm) was incubated at 43°C alone, with IP-HSP60 (OE-NC), IP-HSP60 (OE-NOXA), or BSA (non-chaperone control). Aggregation was monitored by light scattering at 360 nm every 30 s for 60 min.

## 2.10 Pharmacological inhibitor panel

CD4^+^ naïve T cells were activated with anti-CD3/CD28 and co-treated with Ctrl-BD-sEVs or Stress-BD-sEVs (10 μg/mL) in the presence of DMSO vehicle (final concentration ≤0.1%), NIM811 (5 μm), BAI1 (5 μm), VBIT-4 (10 μm), or NAC (5 mM) for 72 h. Cytosolic mtDNA (ND1, ND5) was quantified by qPCR after digitonin-based fractionation. STING pathway activation (p-TBK1, TBK1, p-IRF3, IRF3) was assessed by Western blotting. Treg differentiation was analysed by flow cytometry, and IFN-β secretion was measured by ELISA.

## 2.11 L1CAM functional depletion and brain-sEV functional equivalence

For L1CAM functional depletion, plasma sEVs from stressed mice were precipitated with ExoQuick, incubated with biotinylated anti-L1CAM antibody (Origene, OTI3G1) and streptavidin magnetic beads. The L1CAM^+^ pellet (BD-sEVs) and L1CAM-depleted flow-through (concentrated by ultrafiltration) were applied to CD4^+^ naïve T cells at equal protein input (10 μg/mL) for 72 h. Treg differentiation, NOXA/STING Western blot, cytosolic mtDNA, IFN-β ELISA, and sEV marker validation (CD63, TSG101, L1CAM, synaptophysin, NeuN) were performed.

For brain-sEV functional equivalence, sEVs were isolated directly from whole brain tissue of stressed mice via differential centrifugation and density gradient purification. Plasma L1CAM^+^ BD-sEVs and brain-sEVs were compared side-by-side at equal protein input (10 μg/mL) for effects on Treg differentiation, NOXA/STING signalling, and cytosolic mtDNA release.
