# Supplementary material for: Chronic stress‐induced brain‐derived small extracellular vesicles promote colorectal cancer through Treg differentiation
Source: Clin Transl Med. 2026 Aug 21;16(8):e70756. doi: 10.1002/ctm2.70756 (PMC13494519; doi:10.1002/ctm2.70756)
Supplement: Supplementary file 2 [file CTM2-16-e70756-s002.docx]

# Supplementary Table

## Supplementary Table 1. Baseline characteristics of the retrospective cohort.

| **Characteristic** | **Category** | **Total (n=67)(%)** |
| --- | --- | --- |
| Age, years | mean ± SD | 59.9 ± 13.5 |
|  | range | 26-83 |
| Sex | Male | 40 (59.7) |
|  | Female | 27 (40.3) |
| Differentiation grade | well | 7 (10.4) |
|  | well-moderate | 8 (11.9) |
|  | moderate | 38 (56.7) |
|  | moderate-poor | 7 (10.4) |
|  | poor | 7 (10.4) |
| T category | T1 | 2 (3.0) |
|  | T2 | 2 (3.0) |
|  | T3 | 7 (10.4) |
|  | T4 | 56 (83.6) |
| N category | N0 | 33 (49.3) |
|  | N1 | 26 (38.8) |
|  | N2 | 8 (11.9) |
| M category | M0 | 49 (73.1) |
|  | M1 | 17 (25.4) |
|  | Mx | 1 (1.5) |
| TNM stage | I | 5 (7.5) |
|  | II | 24 (35.8) |
|  | III | 22 (32.8) |
|  | IV | 16 (23.9) |
|  | Unknown | 0 (0.0) |
| Treatment regimen | Surgery only | 4 (6.0) |
|  | Surgery + Observation | 3 (4.5) |
|  | Surgery + Fluoropyrimidine Monotherapy | 1 (1.5) |
|  | Surgery + Adjuvant Chemotherapy | 41 (61.2) |
|  | Surgery + Palliative Systemic Chemotherapy | 18 (26.9) |
| Adjuvant chemo regimen | CAPEOX 3 months | 19 (28.4) |
|  | CAPEOX 6 months | 22 (32.8) |
|  | Capecitabine | 1 (1.5) |
| Chemo regimen | Capecitabine monotherapy | 1 (1.5) |
|  | Capecitabine + Bevacizumab | 2 (3.0) |
|  | FOLFOX + Bevacizumab | 16 (23.9) |
| Survival status | Alive | 57 (85.1) |
|  | Dead | 10 (14.9) |

Values are presented as mean ± SD, n (%), or range. Percentages may not sum to 100% due to rounding.

## Supplementary Table 2. Comparison of baseline characteristics by HAMA score groups in the prospective cohort.

| **Characteristic** | **Category** | **Total (n=37)** | **HAMA < 29 (n=16)** | **HAMA ≥ 29 (n=21)** | ***P*-value** |
| --- | --- | --- | --- | --- | --- |
| HAMA score | mean ± SD | 29.8 ± 9.0 | 21.8 ± 4.1 | 35.9 ± 6.7 | – |
|  | range | 15-48 | 15-28 | 29-48 | – |
| Age, years | mean ± SD | 60.6 ± 17.3 | 64.9 ± 15.6 | 57.3 ± 18.2 | 0.184 |
| Age group | <60 | 14 (37.8) | 4 (25.0) | 10 (47.6) | 0.288 |
|  | >=60 | 23 (62.2) | 12 (75.0) | 11 (52.4) | – |
| BMI, kg/m2 | mean ± SD | 21.2 ± 3.0 | 21.2 ± 2.5 | 21.3 ± 3.3 | 0.870 |
| BMI group | <24 | 29 (78.4) | 13 (81.2) | 16 (76.2) | 1.000 |
|  | >=24 | 8 (21.6) | 3 (18.8) | 5 (23.8) | – |
| Sex | Female | 18 (48.6) | 10 (62.5) | 8 (38.1) | 0.255 |
|  | Male | 19 (51.4) | 6 (37.5) | 13 (61.9) | – |
| Tumor differentiation | G2 | 23 (62.2) | 14 (87.5) | 9 (42.9) | 0.016* |
|  | G3 | 11 (29.7) | 1 (6.2) | 10 (47.6) | – |
|  | Miss | 3 (8.1) | 1 (6.2) | 2 (9.5) | – |
| Cancer stage | I-II | 24 (64.9) | 14 (87.5) | 10 (47.6) | 0.030* |
|  | III-IV | 13 (35.1) | 2 (12.5) | 11 (52.4) | – |
| T category | T1 | 7 (18.9) | 5 (31.2) | 2 (9.5) | 0.323 |
|  | T2 | 8 (21.6) | 4 (25.0) | 4 (19.0) | – |
|  | T3 | 9 (24.3) | 3 (18.8) | 6 (28.6) | – |
|  | T4 | 9 (24.3) | 2 (12.5) | 7 (33.3) | – |
|  | Tx | 1 (2.7) | 0 (0.0) | 1 (4.8) | – |
| N category | N0 | 29 (78.4) | 16 (100.0) | 13 (61.9) | 0.020* |
|  | N1 | 7 (18.9) | 0 (0.0) | 7 (33.3) | – |
|  | N2 | 1 (2.7) | 0 (0.0) | 1 (4.8) | – |
| M category | M0 | 1 (2.7) | 1 (6.2) | 0 (0.0) | 0.354 |
|  | M1 | 1 (2.7) | 0 (0.0) | 1 (4.8) | – |
|  | Mx/Other | 35 (94.6) | 15 (93.8) | 20 (95.2) | – |
| Treatment regimen | Surgery only | 13 (35.1) | 9 (56.2) | 4 (19.0) | 0.015* |
|  | Surgery + Observation | 4 (10.8) | 3 (18.8) | 1 (4.8) | – |
|  | Surgery + Adjuvant Chemotherapy | 16 (43.2) | 4 (25.0) | 12 (57.1) | – |
|  | Surgery + Palliative Systemic Chemotherapy | 4 (10.8) | 0 (0.0) | 4 (19.0) | – |
| Adjuvant chemo regimen | CAPEOX 3 months | 14 (37.8) | 4 (25.0) | 10 (47.6) | – |
|  | CAPEOX 6 months | 2 (5.4) | 0 (0.0) | 2 (9.5) | – |
|  | Capecitabine | 0 (0.0) | 0 (0.0) | 0 (0.0) | – |
| Chemo regimen | FOLFOX + Bevacizumab | 4 (10.8) | 0 (0.0) | 4 (19.0) | – |
|  | Capecitabine monotherapy | 1 (2.7) | 0 (0.0) | 1 (4.8) | – |

Values are presented as mean ± SD or n (%). HAMA, Hamilton Anxiety Rating Scale; SD, standard deviation.

*P*-values were calculated using χ² test or Fisher’s exact test for categorical variables and Student’s t-test or Mann–Whitney U test for continuous variables, as appropriate.

**P* < 0.05; ***P* < 0.01; ****P* < 0.001.

## Supplementary Table 3. Correlation between anxiety scores and clinical characteristics.

| **Retrospective cohort(n=67)** | | | | | | | | | | | |
| --- | --- | --- | --- | --- | --- | --- | --- | --- | --- | --- | --- |
| **Characteristic** | **NOXA low** | **NOXA high** | ***P* value** |  | **FOXP3 low** | **FOXP3 high** | ***P* value** |  | **FOXP3+NOXA low** | **FOXP3+NOXA high** | ***P* value** |
|  |  |  |  |  |  |  |  |  |  |  |  |
| *Age* |  |  |  |  |  |  |  |  |  |  |  |
| < 60 years | 19 | 14 |  |  | 23 | 10 |  |  | 19 | 14 |  |
| ≥ 60 years | 14 | 20 | 0.2722 |  | 16 | 18 | 0.1030 |  | 14 | 20 | 0.2722 |
| *Sex* |  |  |  |  |  |  |  |  |  |  |  |
| Female | 13 | 14 |  |  | 14 | 13 |  |  | 12 | 15 |  |
| Male | 20 | 20 | 1.0000 |  | 25 | 15 | 0.5390 |  | 21 | 19 | 0.6908 |
| *Differentiation* |  |  |  |  |  |  |  |  |  |  |  |
| Well-moderate | 6 | 9 |  |  | 4 | 11 |  |  | 2 | 13 |  |
| Moderate | 23 | 15 |  |  | 27 | 11 |  |  | 25 | 13 |  |
| Poor | 4 | 10 | 0.0888 |  | 8 | 6 | 0.0128 * |  | 6 | 8 | 0.002 * |
| *T category* |  |  |  |  |  |  |  |  |  |  |  |
| T1-T2 | 2 | 2 |  |  | 3 | 1 |  |  | 3 | 1 |  |
| T3 | 4 | 3 |  |  | 4 | 3 |  |  | 4 | 3 |  |
| T4 | 27 | 29 | 0.9051 |  | 32 | 24 | 0.7815 |  | 26 | 30 | 0.4931 |
| *N category* |  |  |  |  |  |  |  |  |  |  |  |
| N0 | 14 | 19 |  |  | 20 | 13 |  |  | 19 | 14 |  |
| N1 | 15 | 11 |  |  | 14 | 12 |  |  | 11 | 15 |  |
| N2 | 4 | 4 | 0.5070 |  | 5 | 3 | 0.8428 |  | 3 | 5 | 0.3949 |
| *M category* |  |  |  |  |  |  |  |  |  |  |  |
| M0 | 22 | 27 |  |  | 28 | 21 |  |  | 25 | 24 |  |
| M1 | 10 | 7 |  |  | 11 | 6 |  |  | 8 | 9 |  |
| Mx | 1 | 0 | 0.3633 |  | 0 | 1 | 0.4251 |  | 0 | 1 | 0.5873 |
| *AJCC stage* |  |  |  |  |  |  |  |  |  |  |  |
| I-II | 12 | 16 |  |  | 16 | 12 |  |  | 15 | 13 |  |
| III-IV | 21 | 18 | 0.5224 |  | 23 | 16 | 1.0000 |  | 18 | 21 | 0.7254 |
| *Treatment* |  |  |  |  |  |  |  |  |  |  |  |
| Surgery/Observation | 3 | 4 |  |  | 5 | 2 |  |  | 5 | 2 |  |
| Surgery + Adj Chemo | 19 | 23 |  |  | 23 | 19 |  |  | 20 | 22 |  |
| Surgery + Pall Chemo | 11 | 7 | 0.4971 |  | 11 | 7 | 0.6803 |  | 8 | 10 | 0.4519 |
| *Survival* |  |  |  |  |  |  |  |  |  |  |  |
| Alive | 28 | 29 |  |  | 34 | 23 |  |  | 28 | 29 |  |
| Dead | 5 | 5 | 1.0000 |  | 5 | 5 | 0.7306 |  | 5 | 5 | 1.0000 |
| *Data are presented as n. P values were calculated using Chi-square test or Fisher's exact test as appropriate. * P < 0.05.* | | | | | | | | | | | |

| **Prospective cohort(n=37)** | | | | | | | | | | | | | | | |
| --- | --- | --- | --- | --- | --- | --- | --- | --- | --- | --- | --- | --- | --- | --- | --- |
| **Characteristic** | **HAMA low** | **HAMA high** | ***P* value** |  | **NOXA low** | **NOXA high** | ***P* value** |  | **FOXP3 low** | **FOXP3 high** | ***P* value** |  | **FOXP3+NOXA low** | **FOXP3+NOXA high** | ***P* value** |
|  |  |  |  |  |  |  |  |  |  |  |  |  |  |  |  |
| *Age* |  |  |  |  |  |  |  |  |  |  |  |  |  |  |  |
| < 60 years | 4 | 10 |  |  | 7 | 7 |  |  | 6 | 8 |  |  | 8 | 6 |  |
| ≥ 60 years | 12 | 11 | 0.1908 |  | 12 | 11 | 1.0000 |  | 13 | 10 | 0.6402 |  | 11 | 12 | 0.8330 |
| *Sex* |  |  |  |  |  |  |  |  |  |  |  |  |  |  |  |
| Female | 10 | 8 |  |  | 12 | 6 |  |  | 11 | 7 |  |  | 10 | 8 |  |
| Male | 6 | 13 | 0.2545 |  | 7 | 12 | 0.1375 |  | 8 | 11 | 0.4082 |  | 9 | 10 | 0.8658 |
| *BMI* |  |  |  |  |  |  |  |  |  |  |  |  |  |  |  |
| < 24 | 13 | 16 |  |  | 15 | 14 |  |  | 14 | 15 |  |  | 14 | 15 |  |
| ≥ 24 | 3 | 5 | 1.0000 |  | 4 | 4 | 1.0000 |  | 5 | 3 | 0.6928 |  | 5 | 3 | 0.6928 |
| *Differentiation* |  |  |  |  |  |  |  |  |  |  |  |  |  |  |  |
| G2 | 14 | 9 |  |  | 12 | 11 |  |  | 15 | 8 |  |  | 14 | 9 |  |
| G3 | 1 | 10 |  |  | 5 | 6 |  |  | 2 | 9 |  |  | 4 | 7 |  |
| Miss | 1 | 2 | 0.0161 * |  | 2 | 1 | 0.8021 |  | 2 | 1 | 0.0318 * |  | 1 | 2 | 0.3307 |
| *T category* |  |  |  |  |  |  |  |  |  |  |  |  |  |  |  |
| T0-T2 | 11 | 7 |  |  | 11 | 7 |  |  | 10 | 8 |  |  | 12 | 6 |  |
| T3 | 3 | 6 |  |  | 3 | 6 |  |  | 4 | 5 |  |  | 3 | 6 |  |
| T4 | 2 | 8 | 0.0862 |  | 5 | 5 | 0.3939 |  | 5 | 5 | 0.8579 |  | 4 | 6 | 0.1849 |
| *N category* |  |  |  |  |  |  |  |  |  |  |  |  |  |  |  |
| N0 | 16 | 13 |  |  | 17 | 12 |  |  | 16 | 13 |  |  | 18 | 11 |  |
| N1-N2 | 0 | 8 | 0.006 * |  | 2 | 6 | 0.1245 |  | 3 | 5 | 0.4470 |  | 1 | 7 | 0.0188 * |
| *M category* |  |  |  |  |  |  |  |  |  |  |  |  |  |  |  |
| M0/Mx | 16 | 20 |  |  | 19 | 17 |  |  | 18 | 18 |  |  | 19 | 17 |  |
| M1 | 0 | 1 | 1.0000 |  | 0 | 1 | 0.4865 |  | 1 | 0 | 1.0000 |  | 0 | 1 | 0.4865 |
| *AJCC stage* |  |  |  |  |  |  |  |  |  |  |  |  |  |  |  |
| I-II | 12 | 6 |  |  | 10 | 8 |  |  | 9 | 9 |  |  | 11 | 7 |  |
| III-IV | 4 | 15 | 0.008 * |  | 9 | 10 | 0.8658 |  | 10 | 9 | 1.0000 |  | 8 | 11 | 0.4082 |
| *Treatment* |  |  |  |  |  |  |  |  |  |  |  |  |  |  |  |
| Surgery/Observation | 12 | 5 |  |  | 8 | 9 |  |  | 8 | 9 |  |  | 10 | 7 |  |
| Surgery + Adj Chemo | 4 | 12 |  |  | 10 | 6 |  |  | 9 | 7 |  |  | 8 | 8 |  |
| Surgery + Pall Chemo | 0 | 4 | 0.006 * |  | 1 | 3 | 0.3618 |  | 2 | 2 | 0.8685 |  | 1 | 3 | 0.4715 |
| *Data are presented as n. P values were calculated using Chi-square test or Fisher's exact test as appropriate. *P < 0.05. HAMA: ≧29 high vs <29 low score groups; biomarker expression groups by prospective cohort median cutoff.* | | | | | | | | | | | | | | | |

## Supplementary Table 4. Antibodies used in this study.

*Western blot / Co-IP / Subcellular fractionation — Primary antibodies*

| **Antibody** | **Source** | **Identifier** |
| --- | --- | --- |
| Anti-NOXA (PMAIP1) | Santa Cruz Biotechnology | sc-56169 |
| Anti-β-actin | Proteintech | 20536-1-AP |
| Anti-Flag (DYKDDDDK Tag) | Cell Signaling Technology | 8146S |
| Anti-His (6×His Tag) | Cell Signaling Technology | 2366S |
| Anti-HSP60 (HSPD1) | Cell Signaling Technology | 12165S |
| Anti-phospho-TBK1/NAK (Ser172) (D52C2) | Cell Signaling Technology | 5483 |
| Anti-TBK1/NAK | Proteintech | 67211-1-Ig |
| Anti-phospho-IRF-3 (Ser396) (4D4G) | Cell Signaling Technology | 29047 |
| Anti-IRF-3 | Proteintech | 11312-1-AP |
| Anti-FOXP3 | Proteintech | 22228-1-AP |
| Anti-CD63 | Proteintech | 25682-1-AP |
| Anti-TSG101 | Proteintech | 28283-1-AP |
| Anti-L1CAM (CD171) | Proteintech | 31534-1-AP |
| Anti-synaptophysin (SYP) | Proteintech | 17785-1-AP |
| Anti-NeuN (RBFOX3) | Proteintech | 26975-1-AP |
| Anti-GFAP | Proteintech | 60190-1-Ig |
| Anti-p75-NTR (NGFR/CD271) | Proteintech | 55014-1-AP |
| Anti-cytochrome c (CYCS) | Proteintech | 10993-1-AP |
| Anti-COX IV (COX4I1) | Proteintech | 11242-1-AP |
| Anti-MCL-1 | Proteintech | 16225-1-AP |
| Anti-poly-ubiquitin (clone FK2) | Enzo Life Sciences | BML-PW8810 |
| Anti-normal rabbit IgG (IP control) | Cell Signaling Technology | 2729S |

*Secondary antibodies*

| **Antibody** | **Source** | **Identifier** |
| --- | --- | --- |
| HRP-conjugated Goat Anti-Rabbit IgG (H+L) | Beyotime | A0208 |
| HRP-conjugated Goat Anti-Mouse IgG (H+L) | Beyotime | A0216 |

*Flow cytometry antibodies (mouse)*

| **Antibody** | **Source** | **Identifier** |
| --- | --- | --- |
| Anti-mouse CD45 FITC | eBioscience | 11-0451-82 |
| Anti-mouse CD3 PE/Cyanine5 | BioLegend | 100273 |
| Anti-mouse CD4 PE-Cy7 | BioLegend | 116016 |
| Anti-mouse CD4 Brilliant Violet 650 | BioLegend | 100545 |
| Anti-mouse CD8 Brilliant Violet 785 | BioLegend | 100749 |
| Anti-mouse CD25 PE | BioLegend | 101904 |
| Anti-mouse FOXP3 Alexa Fluor 647 | BD Biosciences | 560401 |
| Anti-mouse CD80 PE-Cyanine7 | eBioscience | 25-0801-82 |
| Anti-mouse CD80 PE | BioLegend | 104708 |
| Anti-mouse MHC-II (I-A/I-E) PE | eBioscience | 12-5321-82 |
| Anti-mouse I-A/I-E PE/Cyanine7 | BioLegend | 107630 |
| Anti-mouse CD11b Elab Fluor Violet 610 | Elabscience | E-AB-F1081T |
| Anti-mouse CD11c Elab Fluor Violet 500 | Elabscience | E-AB-F0991R |
| Anti-mouse CD19 Elab Fluor Violet 450 | Elabscience | E-AB-F0986Q |
| Anti-mouse CD206 Alexa Fluor 647 | Starter Bio | S0B5331 |
| Anti-mouse LY-6G Alexa Fluor 700 | Starter Bio | S0B5116 |
| Anti-mouse CD45 FITC | BioLegend | 157214 |
| Anti-mouse IL-17 PE/Dazzle 594 | BioLegend | 506937 |
| Anti-mouse IL-4 PerCP/Cyanine5.5 | BioLegend | 504123 |
| Anti-mouse IFN-γ FITC (clone XMG1.2) | BioLegend | 505806 |
| Anti-mouse Granzyme B APC (clone QA16A02) | BioLegend | 372204 |
| Anti-mouse PD-1 PE (clone 29F.1A12) | BioLegend | 135206 |
| Anti-mouse TIM-3 PE (clone RMT3-23) | BioLegend | 119704 |
| Anti-mouse CTLA-4 PE (clone UC10-4B9) | BioLegend | 106306 |
| Anti-human CD4 PerCP-Cyanine7 | eBioscience | 25-0048-42 |
| Anti-human CD25 PE | eBioscience | 12-0259-42 |
| Anti-human FOXP3 APC | eBioscience | 17-4776-42 |

| **Reagent** | **Source** | **Identifier** |
| --- | --- | --- |
| Zombie NIR Fixable Viability Dye | BioLegend | 423105 |

| **Antibody** | **Source** | **Identifier** |
| --- | --- | --- |
| Anti-CD4 | Proteintech | 67786-1-Ig |
| Anti-FOXP3 | Proteintech | 22228-1-AP |
| Anti-NOXA (PMAIP1) | Proteintech | 11415-1-AP |
| Anti-CD8 | Proteintech | 66868-1-Ig |
| Anti-PD-L1 (CD274) | Proteintech | 66248-1-Ig |
| Anti-PD-1 (PDCD1) | Proteintech | 66220-1-Ig |

*sEV enrichment antibodies (biotinylated) / In vivo depletion / T-cell activation*

| **Antibody / Reagent** | **Source** | **Identifier** |
| --- | --- | --- |
| Biotinylated anti-mouse L1CAM (clone OTI3G1) | Origene | TA801177 |
| Biotinylated anti-mouse p75-NTR (NGFR) | R&D Systems | BAM1157 |
| Purified anti-mouse CD3ε (clone 145-2C11) | BioLegend | 100339 |
| Purified anti-mouse CD28 (clone 37.51) | BioLegend | 102115 |
| Purified anti-human CD3 (clone OKT3) | BioLegend | 317326 |
| Purified anti-human CD28 (clone CD28.2) | BioLegend | 377204 |
| Anti-mouse CD25 (clone PC61, in vivo depletion) | BioLegend | 102032 |
| Anti-mouse isotype control IgG (in vivo) | BioLegend | 400634 |

*Recombinant cytokines (for T-cell activation)*

| **Cytokine** | **Source** | **Identifier** |
| --- | --- | --- |
| Recombinant Human IL-2 | PeproTech | 200-02 |
| Recombinant Mouse IL-2 | PeproTech | 212-12 |

## Supplementary Table 5. Reagents used in this study.

| **Kit / Assay** | **Source** | **Identifier** |
| --- | --- | --- |
| RevertAid First-Strand cDNA Synthesis Kit | Thermo Fisher Scientific | K1622 |
| RNA Purification Kit | EZBioscience | B0004D |
| SYBR qPCR Master Mix | Tsingke | TSE501 |
| Human Naïve CD4+ T Cell Isolation Kit II | Miltenyi Biotec | 130-094-131 |
| Mouse CD4+ T Cell Isolation Kit II | Miltenyi Biotec | 130-104-454 |
| CD4+CD25+ Regulatory T Cell Isolation Kit, human | Miltenyi Biotec | 130-091-301 |
| CD4+CD25+ Regulatory T Cell Isolation Kit, mouse | Miltenyi Biotec | 130-091-041 |
| Annexin V-FITC Apoptosis Detection Kit | BioLegend | 640905 |
| Foxp3 Transcription Factor Fixation/Permeabilization Kit | eBioscience | 00-5523-00 |
| EndoFree Maxi Plasmid Kit | TIANGEN | DP117 |
| Cell Mitochondrial Isolation Kit | Beyotime | C3601 |
| Cell Lysis Buffer for Western and IP | Beyotime | P0013 |
| BCA Protein Assay Kit | Beyotime | P0010 |
| Enhanced BCA Protein Assay Kit | Beyotime | P0010S |
| ExoQuick Precipitation Kit | System Biosciences (SBI) | EXOQ5A-1 |
| Bulge-Loop miRNA qRT-PCR Starter Kit | RiboBio | C10211-2 |
| PrimeScript RT-PCR Kit | TaKaRa | RR014A |
| Human mtDNA Monitoring Primer Set | TaKaRa | 7246 |
| Human TGF-β1 ELISA Kit | Dakewe | 1117102 |
| Human IL-10 ELISA Kit | Dakewe | 1111002 |
| Mouse IFN-β ELISA Kit | R&D Systems | MIFNB0 |
| CCK-8 Cell Proliferation/Viability Assay Kit | Beyotime | C0037 |

| **Protein / Cytokine** | **Source** | **Identifier** |
| --- | --- | --- |
| Recombinant Human TGF-β1 | PeproTech | 100-21 |
| Recombinant Human IL-10 | PeproTech | 200-10 |

| **Chemical / Drug / Inhibitor** | **Source** | **Identifier** |
| --- | --- | --- |
| PEI (Polyethylenimine) | Polysciences | 23966 |
| Polybrene (Hexadimethrine bromide) | Sigma-Aldrich | TR-1003-G |
| RIPA Lysis Buffer | Beyotime | P0038 |
| CFSE (Carboxyfluorescein succinimidyl ester) | Invitrogen | C34554 |
| Propidium Iodide (PI) | Beyotime | ST511 |
| Protease Inhibitor Cocktail | Beyotime | P1008 |
| BD GolgiStop Protein Transport Inhibitor | BD Biosciences | 554724 |
| Ionomycin | Absin | abs9108-1mg |
| Phorbol 12-myristate 13-acetate (PMA) | Absin | abs9107-1mg |
| Mouse 1× Lymphocyte Separation Medium | Dakewe | 7211011 |
| Human 1× Lymphocyte Separation Medium | Dakewe | 7111012 |
| NIM811 (mPTP inhibitor) | Selleck Chemicals | S7517 |
| BAI1 (BAX inhibitor) | Selleck Chemicals | S8865 |
| VBIT-4 (VDAC oligomerization inhibitor) | Selleck Chemicals | S3544 |
| N-Acetylcysteine (NAC, ROS scavenger) | Selleck Chemicals | S1623 |
| MG132 (proteasome inhibitor) | Selleck Chemicals | S2619 |
| CCCP (mitochondrial uncoupler) | Selleck Chemicals | S6494 |
| Cycloheximide (CHX) | Selleck Chemicals | S7418 |
| Propranolol hydrochloride | Selleck Chemicals | S1738 |
| Mifepristone (RU-486) | Selleck Chemicals | S1746 |
| Muscimol (GABAA agonist) | Selleck Chemicals | S3674 |
| Heparin sodium salt | Sigma-Aldrich | H3149 |
| Protease K | Sigma-Aldrich | P6556 |
| RNase A | Sigma-Aldrich | R6513 |
| RNase Inhibitor (RNasin) | Promega | N2611 |
| PMSF (phenylmethylsulfonyl fluoride) | Beyotime | ST506 |
| Digitonin | Sigma-Aldrich | D141 |
| DMSO (Dimethyl sulfoxide) | Sigma-Aldrich | D2650 |
| Isoflurane | RWD Life Science | R510-22 |
| CellEvent Caspase-3/7 Green Detection Reagent | Invitrogen | C10423 |
| JC-1 Mitochondrial Membrane Potential Probe | Beyotime | C2006 |
| DCFH-DA (ROS detection probe) | Beyotime | S0033 |
| ATP Bioluminescence Assay Kit | Beyotime | S0026 |
| Citrate Synthase (CS) from porcine heart | Sigma-Aldrich | C3260 |
| Streptavidin Magnetic Beads | Thermo Fisher Scientific | 88817 |
| miRNA mimics (miR-342-3p, miR-381-3p, miR-15a-5p, mimic NC) | RiboBio | custom synthesis |
| Antagomirs (antagomir-miR-342-3p, -miR-381-3p, -miR-15a-5p, antagomir-NC) | RiboBio | custom synthesis |

*Cell culture reagents*

| **Reagent** | **Source** | **Identifier** |
| --- | --- | --- |
| DMEM (Dulbecco's Modified Eagle Medium) | Gibco | C11995500BT |
| RPMI 1640 Medium | Gibco | C11875500BT |
| Fetal Bovine Serum (FBS) | Gibco | 10099-141 |
| Penicillin-Streptomycin (10,000 U/mL) | Gibco | 15140-122 |
| 0.25% Trypsin-EDTA | Gibco | 25200-056 |

*Western blot / Molecular biology reagents*

| **Reagent** | **Source** | **Identifier** |
| --- | --- | --- |
| TRIzol Reagent | Invitrogen | 15596018 |
| PVDF Membrane (0.45 μm) | Millipore | IPVH00010 |
| ECL Substrate (Femto-pico) | FDbio | FD8030 |
| 10% SDS-PAGE Precast Gel | Invitrogen | NP0301BOX |
| PageRuler Prestained Protein Ladder | Thermo Fisher Scientific | 26616 |
| Non-fat dry milk (blocking) | BD Difco | 232100 |
| TWEEN 20 | Sigma-Aldrich | P1379 |
| Glycine | Sigma-Aldrich | G8898 |
| Tris base | Sigma-Aldrich | T1503 |
| Sodium dodecyl sulfate (SDS) | Sigma-Aldrich | L3771 |

*miRNA qPCR primers (Bulge-Loop, RiboBio)*

| **Primer / Probe** | **Source** | **Identifier** |
| --- | --- | --- |
| hsa-miR-342-3p Bulge-Loop miRNA qRT-PCR Primer | RiboBio | 242560000601-1 |
| hsa-miR-15a-5p Bulge-Loop miRNA qRT-PCR Primer | RiboBio | MQPS0000698-1 |
| hsa-miR-381-3p Bulge-Loop miRNA qRT-PCR Primer | RiboBio | MQPS0000120-1 |
| U6 snRNA Bulge-Loop qRT-PCR Primer (internal control) | RiboBio | MQPS0000002-1 |

*Plasmids and cell lines*

| **Plasmid / Cell Line** | **Source** | **Identifier** |
| --- | --- | --- |
| pSin-EF2-Puro (lentiviral backbone) | Addgene | 16541 |
| psPAX2 (lentiviral packaging) | Addgene | 12260 |
| pMD2.G (VSV-G envelope) | Addgene | 12259 |
